# Embryonic mammary signature subsets are activated in *Brca1^-/- ^*and basal-like breast cancers

**DOI:** 10.1186/bcr3403

**Published:** 2013-03-18

**Authors:** Marketa Zvelebil, Erik Oliemuller, Qiong Gao, Olivia Wansbury, Alan Mackay, Howard Kendrick, Matthew J Smalley, Jorge S Reis-Filho, Beatrice A Howard

**Affiliations:** 1 Division of Breast Cancer Research, Breakthrough Breast Cancer Research Centre, The Institute of Cancer Research, 237 Fulham Road, London SW3 6JB, UK; 2 European Cancer Stem Cell Research Institute and Cardiff School of Biosciences, Cardiff University, Museum Avenue, Cardiff CF10 3AX, UK; 3 Department of Pathology, Memorial Sloan-Kettering Cancer Center, New York, NY 10065, USA

## Abstract

**Introduction:**

Cancer is often suggested to result from development gone awry. Links between normal embryonic development and cancer biology have been postulated, but no defined genetic basis has been established. We recently published the first transcriptomic analysis of embryonic mammary cell populations. Embryonic mammary epithelial cells are an immature progenitor cell population, lacking differentiation markers, which is reflected in their very distinct genetic profiles when compared with those of their postnatal descendents.

**Methods:**

We defined an embryonic mammary epithelial signature that incorporates the most highly expressed genes from embryonic mammary epithelium when compared with the postnatal mammary epithelial cells. We looked for activation of the embryonic mammary epithelial signature in mouse mammary tumors that formed in mice in which *Brca1 *had been conditionally deleted from the mammary epithelium and in human breast cancers to determine whether any genetic links exist between embryonic mammary cells and breast cancers.

**Results:**

Small subsets of the embryonic mammary epithelial signature were consistently activated in mouse *Brca1*^-/- ^tumors and human basal-like breast cancers, which encoded predominantly transcriptional regulators, cell-cycle, and actin cytoskeleton components. Other embryonic gene subsets were found activated in non-basal-like tumor subtypes and repressed in basal-like tumors, including regulators of neuronal differentiation, transcription, and cell biosynthesis. Several embryonic genes showed significant upregulation in estrogen receptor (ER)-negative, progesterone receptor (PR)-negative, and/or grade 3 breast cancers. Among them, the transcription factor, *SOX11*, a progenitor cell and lineage regulator of nonmammary cell types, is found highly expressed in some *Brca1^-/- ^*mammary tumors. By using RNA interference to silence *SOX11 *expression in breast cancer cells, we found evidence that SOX11 regulates breast cancer cell proliferation and cell survival.

**Conclusions:**

Specific subsets of embryonic mammary genes, rather than the entire embryonic development transcriptomic program, are activated in tumorigenesis. Genes involved in embryonic mammary development are consistently upregulated in some breast cancers and warrant further investigation, potentially in drug-discovery research endeavors.

## Introduction

The notion that some cancers may arise because of the reactivation of embryonic developmental programs was first proposed in the 19th century. Among the proponents of this idea was Rudolf Virchow, who recognized elements of embryonic development in cancers. Virchow coined the term "teratoma" to describe tumors containing differentiated elements of the three embryonic germ layers and also suggested that cancers arise from embryo-like cells [1]. Lobstein and Cohnheim [2] also noted similarities between embryogenesis and the biology of cancer cells and put forward the hypothesis that tumorigenesis recapitulates aspects of development [2]. During organ formation, cells proliferate, migrate, and invade into adjacent tissues to produce highly organized tissues, and these same cellular processes are used during carcinogenesis, which results in the formation of relatively organized populations of abnormal cells, which comprise tumors. Therefore, it has been suggested that some tumors arise from reactivation of embryonic developmental programs in postnatal tissues.

Two of the most common breast cancer-driver mutations, which confer clonal selective advantage on cancer cells and are causally implicated in oncogenesis, are found in *GATA3 *and *TBX3*, which are genes that have been shown to be required for embryonic mammary development [3-5]. Many other signaling pathways have also been implicated in both embryonic mammary morphogenesis and carcinogenesis, providing support for the contention that neoplastic and immature tissues share important similarities and that organ development and primary tumor formation are likely to be underpinned by common mechanisms [6]. Newly identified cancer stem cells in skin, gut, and brain are very similar to healthy stem cells responsible for growing and renewing tissue in the body, highlighting the need for further understanding of the normal mammary progenitor cells and their potential links to cancer, as tumors may develop from progenitor-like cells from diverse stages of cellular differentiation [7-9].

Recently we completed a transcriptomic analysis of embryonic mouse mammary primordial cells, the first such study of separated embryonic mammary epithelial and mammary mesenchymal cell populations [10]. These two cell populations interact in a complex, reciprocal manner as the mammary primordium forms during embryogenesis. Recent data from cell-lineage tracing studies suggest that embryonic mammary cells are the only cell populations that are truly multipotent *in vivo *[11] . Embryonic mammary epithelial cells are an immature cell population, lacking differentiation markers, which is reflected in their very distinct genetic profiles when compared with those of their postnatal descendents [10].

In this study, we explored the hypothesis that reactivation of embryonic developmental programs in mature breast cells promotes tumor formation. We defined an embryonic mammary signature to incorporate the most highly expressed genes from the embryonic epithelium during organ formation when compared with the postnatal mammary epithelial cells and compared them with gene signatures of breast cancers. We found reactivation of small modules of embryonic mammary epithelial genes within mouse *Brca1^-/- ^*tumors and human basal-like/triple-negative breast cancers. Many embryonic genes are activated across breast cancer datasets, and several are linked to clinical parameters, including hormone-receptor expression, subtype, and grade. We found that embryonic mammary signature activation in breast cancer samples is predictive of breast cancer patient outcome, suggesting clinical relevance. Our studies therefore provide new insights into the association of embryonic signature activation with clinical features of some breast cancers.

## Materials and methods

### Data analysis

Transcriptome analysis on normal mammary populations and tumor RNA profiled with Affymetrix 430 2.0 mouse gene-expression chips was as described [10,12,13]. The microarray data are available in ArrayExpress with accession numbers E-TABM-1099, E-TABM-683, E-TABM-684, and E-TABM-997. Raw Affymetrix.CEL files were normalized and summarized by robust multiarray analysis (RMA) by using the Affy package from BioConductor [14]. Probe sets were used for a multiclass Significance Analysis of Microarrays (SAM) by using a local false-discovery rate of 5% to determine whether their mean expression was different across the three mammary epithelial cell (MEC) subpopulations and three embryonic mammary populations described [10,13]. Probes are considered embryonic-enriched when they have a mean relative abundance of 10-fold or more when compared with the postnatal mammary epithelial samples.

With 799 probe sets shown to distinguish robustly between embryonic mammary epithelium and postnatal mammary cells, normal and tumor samples were clustered by using a Ward algorithm based on Pearson correlation distance. Human orthologues for 689 genes encoded by the 799-probe set were used to cluster human breast cancers in three datasets [15-17] based on Ward clustering with correlation distance. Breast cancer subtypes in the Natrajan [16] and NKI295 [15] datasets were as defined by the research version of PAM50 classification [18]; PAM50 from Parker *et al. *[17] was used to describe subtypes in the UNC337 dataset [17]. The 70-gene prognosis signature was used to classify tumors into poor or good prognosis on the basis of their risk of developing distant metastases within 5 years [15,19].

We tested for presence of clusters and observed hierarchic clustering with two clusters to be the most suitable for our dataset. The agglomerative method of Ward hierarchic clustering, as implemented in the R-package pvclust [20], was used for subsequent analysis. Parameters were set to 10,000 bootstrap replicates, with relative sample sizes set from 0.5 to 1.4, incrementing in steps of 0.1 to determine AU (approximately unbiased) *P *values. Hypergeometric statistical analysis was used to demonstrate that enrichment of embryonic gene activation in mouse tumor and breast cancer datasets was significant.

We used proliferation signatures defined by Ben-Porath *et al. *[21] to designate tumor-associated embryonic genes as proliferative or not. Two additional proliferation signatures, defined, by Desmedt *et al. *[22] and Ghazoui *et al. *[23], provided a list of additional genes to exclude. For Spearman correlation, a cut-off was used to exclude all genes with an absolute correlation > 0.5 with proliferation genes.

From the embryonic and postnatal mammary gene signatures, centroids were defined for 37 genes comprising the nonproliferative embryonic gene signature. Centroid correlation was performed with the NKI295 dataset by using Spearman correlation. The nearest centroid was recorded for every sample, and those with correlation of < 0.1 were assigned to no correlation, whereas those with a correlation ≥ 0.1 were classified as "embryonic." Kaplan-Meier analysis and multivariate Cox proportional hazard regression analysis were carried out with the R survival package. The nonproliferative embryonic gene signature and tumor annotations were tested in models containing various combinations of tumor size, differentiation status, lymph node positivity, ER status, and 70-gene signature, as indicated.

### Pathway and network analysis

Pathway analysis was performed by using functional annotation cluster analysis by using DAVID [24]. An interaction network was generated within ROCK by using genes of interest and visualized by using ROCKscape [25]. Initially, only interactions between selected genes were allowed; this was then extended by allowing one joining gene between two selected genes to form interactions where the genes were not interacting in the first phase.

### Statistical analysis of embryonic mammary genes in tumors

For expression fold-change, genes were submitted in the ROCK resource [25] to identify significant changes in expression between specific groups of tumors. Only studies in which samples were run on the same chip and normalized in the same manner were included. An average fold-change of twofold or more (up or down) was considered a significant fold-change. Results were also verified by using (SAM) analysis tool in ROCK to determine significant changes of expression in subtypes, tumor type, and grade classification. Molecular subtypes were defined by PAM50 [17].

For survival curves, genes with significant expression changes were subjected to Kaplan-Meier plot survival calculation within the ROCK resource. Significant impact on survival was assumed if the χ^2 ^*P *value was < 0.05, or its associated log_2 _rank *P *value was < 0.05.

### Sample collection

All animal work was carried out under UK Home Office project and personal licenses after local ethical approval from The Institute of Cancer Research Ethics Committee and in accordance with local and national guidelines. Embryonic day 12.5 (E12.5) mammary primordia were manually microdissected, and tissue separations were performed as previously described [10].

### Quantitative real-time polymerase chain reaction

Total RNA was extracted from purified populations of two to three independent biologic replicates by using Qiagen RNeasy Micro Plus kit (Qiagen, Hilden, Germany). cDNA synthesis of RNA was carried out by using Quantitect Reverse Transcription kit (Qiagen, Hilden, Germany) and run with TaqMan Array Assay-on-Demand probes (Applied Biosystems, Life Technologies Corporation, Carlsbad, CA, USA). Results were analyzed by using the ^Δ-Δ^Ct method normalized to *Actb*. Total RNA from tumor and mammary samples were reverse transcribed and linearly amplified by using the Ovation Amplification System V2 kit (NuGEN Technologies, San Carlos, CA, USA), as described previously, before Quantitative real-time polymerase chain reaction (qRT-PCR) analysis [10]. The expressions of *SOX11 *in BT474 and BT549 cells were analyzed with qRT-PCR by using TaqMan Gene Expression Assay for *SOX11*, Hs00846583_s1 (Applied Biosystems, Life Technologies Corporation, Carlsbad, CA, USA) combined with FAM and normalized against β-actin, Hs99999903_m1, combined with VIC.

### Immunohistochemistry and whole-mount immunofluorescence

Methods were as previously described [10,26]. Antibodies are listed in Additional file 1A; Sox11 guinea pig antiserum is described [27]; and the specificity of this antibody was previously demonstrated [28]. Transverse cryosections from the forelimb region of *Sox11^-/- ^*embryos were used to demonstrate the specificity of the SOX11 mouse monoclonal antibody MRQ-58 from Cell Marque (Rocklin, CA, USA) in mouse tissue. Negative controls were performed for all antibodies by the omission of primary antibody. Expression at other sites (embryonic brain or skin) was used for positive controls. Representative micrographs of controls are shown in Additional file 1B, C.

### *SOX11 *knockdown in breast cancer cells

BT474 and BT549 cells were transfected with 80 pmol of each *SOX11 *siRNA (siGENOME SMARTpool and four individual siRNAs), control nontargeting siRNA or Cyclophilin control siRNA (Thermo Scientific, Waltham, MA, USA) by using Lipofectamine 2000 (Invitrogen, Life Technologies Corporation, Carlsbad, CA, USA) in Opti-MEM (Gibco, Life Technologies Corporation, Carlsbad, CA, USA) media according to the manufacturer's instructions for 6 hours in a six-well plate, and then incubated with DMEM supplemented with 10% fetal bovine serum.

BT474 cells were lysed with RIPA buffer 72 hours after transfection and subjected to immunoblotting, as previously described [29]. SOX11 expression was detected by using a rabbit monoclonal antibody (Epitomics, Burlingame, CA, USA, clone EPR8192); caspase-3 (R&D Systems, Minneapolis, MN, USA) and cleaved caspase-3 (Cell Signaling Technology, Danvers, MA, USA) were detected by using mouse monoclonal and rabbit polyclonal antibodies.

### *SOX11 *overexpression

The 1 × 10^6 ^BT549 cells were transfected with 3 μg of either pCMV6-AC-GFP plasmid containing the sequence for a fusion protein between SOX11 and GFP (RG220681, Origene Rockville, MD, USA) or a control plasmid containing GFP, pIRES2-EGFP (Clontech, Mountain View, CA, USA), by nucleoporation by using the Amaxa Cell Line Nucleofector kit V (Lonza, Basel, Switzerland) with the T-024 program. The transfection efficiency was evaluated with flow cytometry.

### Cell-viability assays

At 48 hours after transfection, 3,000 BT474 cells or 1,000 BT549 cells were plated per well of a 96-well plate. Cell-growth rates were assessed 24, 48, and 72 hours later by incubating for 2 hours with PrestoBlue Cell Viability Reagent (Life Technologies, Carlsbad, CA, USA). The absorbance obtained at each time point was normalized to the absorbance at 0 hours. Statistical significance was determined by using a two-way ANOVA test followed by a Bonferroni *post hoc *test. The results at 72 hours are presented as the percentage of growth relative to the population transfected with the nontargeting siRNA. Statistical significance was determined by using a 1-way ANOVA test followed by a Bonferroni *post hoc *test.

### Cell-cycle analysis

Cell populations were trypsinized 48 hours after transfection with siRNAs and fixed in 70% ethanol overnight. After a 1-hour incubation with RNase A at 37°C, the cells were stained with 7AAD (eBioscience, San Diego, CA, USA) before they were subjected to FACs analysis by using a BD LSR II flow cytometer and analyzed with the FACSDiva software. Statistical significance was determined by using a one-way ANOVA test followed by a Bonferroni *post hoc *test.

## Results

### Embryonic mammary epithelial cells are estrogen receptor (ER)^-^, progesterone receptor (PR)^-^, and express low levels////of Erbb2

**Figure 1:**
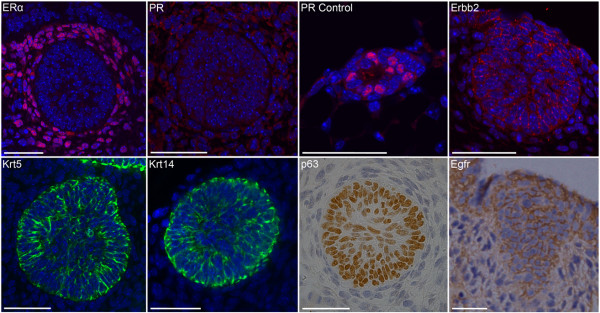
**Embryonic mammary bud epithelial cells share key marker profiles with *Brca1^-/- ^*and basal-like breast cancers**. Many E12.5-stage embryonic mammary bud epithelial cells display a triple-negative profile. Immunofluorescence (IF) with ERα shows stain throughout the mammary mesenchymal tissue and no epithelial stain. IF with PR shows no staining of either mammary epithelial or mesenchymal cells. Control tissue shows staining in some luminal mammary epithelial cells in postnatal tissue. Erbb2 is expressed at low to moderate levels by some embryonic mammary epithelial cells, whereas other cells do not stain. Krt5 and Krt14 are highly expressed by many, but not all, embryonic mammary epithelial cells. All embryonic mammary epithelial cells express p63; many express low to moderate levels of Egfr. Scale bar, 50 μm.

Midgestation embryonic mammary bud epithelial (MBE) cells are ER^-^, PR^- ^and express low to moderate levels of Erbb2 (Figure 1). Many MBE cells express high levels of basal keratins (Krt5, Krt14), Egfr, and all express p63 (Figure 1). MBE cells exhibit marker profiles similar to those used to describe the defining features of triple-negative and basal-like breast cancers and may use similar signaling pathways and networks to underpin key biologic properties of similar cell types found enriched within both populations.

### Subsets of the embryonic mammary signatures are activated in *Brca1^-/- ^*mouse tumors

We defined an embryonic mammary signature based on expression profiles of genes found highly expressed within midgestation (E12.5-stage) embryonic epithelium compared with postnatal mammary epithelial cells described in Additional file 2[10,13]. This signature is distinct from the fetal mammary stem cell signature recently defined by Spike *et al. *[30], which profiled subpopulations of late-gestation (E18.5-stage) mammary cells. Only 12 genes (1.4%) are shared between the two embryonic signatures, which are both defined by enriched expression in embryonic versus postnatal mammary cell populations (see Additional file 2).

**Figure 2:**
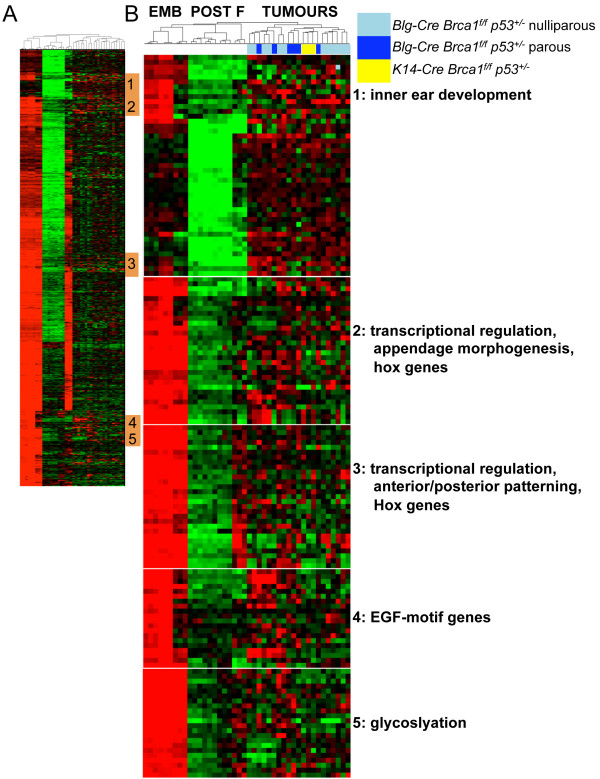
**Subsets of the embryonic mammary epithelial signature are activated in *Brca1^-/- ^*mouse tumors**. **(A) **Unsupervised hierarchic clustering of embryonic epithelial signature expression in *Brca1^-/- ^*mouse mammary tumor dataset. **(B) **Five clusters of *Brca1^-/- ^*tumor-associated embryonic genes and functional annotation. EMB, embryonic mammary cells; POST, postnatal mammary epithelial cells; F, mammary fibroblasts [10]. TUMOURS are *Brca1^-/- ^*mammary tumors from [12].

Next, we interrogated the embryonic-enriched mammary epithelial signature expression in mammary tumors that formed in mouse strains in which *Brca1 *had been deleted in either mammary epithelial luminal progenitors (*Blg-Cre Brca1^f/f ^p53^+/-^*) or in basal cells, including basal stem cells (*K14-Cre Brca1^f/f ^p53^+/-^*) [12], to determine whether the embryonic signature is activated in a validated mouse model of triple-negative breast cancer [31]. Small subsets of the embryonic epithelial signature (123 of 689 genes (18%)) were activated in *Brca1^-/- ^*mouse tumors when the embryonic epithelial signature was used for hierarchic cluster analysis (Figure 2A, B and Additional file 3).

### Subsets of the embryonic mammary signatures are activated in human breast cancers

Because only subsets of the embryonic mammary signature, and not the entire developmental program, appear activated in mouse tumors, we sought to define the genes shared between the embryonic signature and breast cancers across multiple datasets. We reasoned that this strategy should result in the identification of embryonic mammary genes consistently activated in breast cancers that are not normally highly expressed by postnatal mammary epithelial cells.

**Figure 3:**
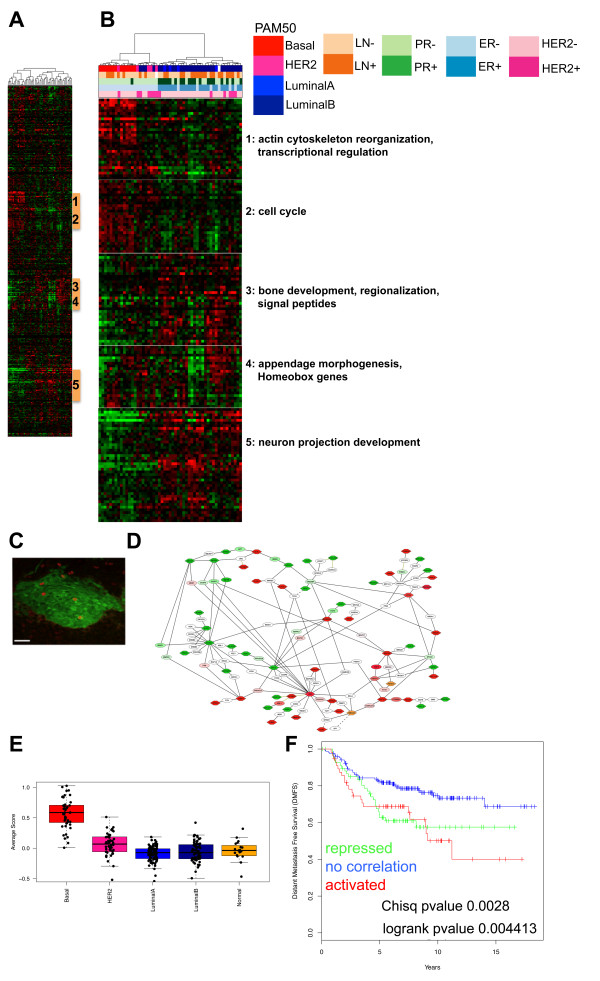
**Subsets of the embryonic epithelial mammary signature are activated in microdissected human breast cancers**. **(A) **Unsupervised hierarchic clustering of embryonic epithelial signature expressed in Natrajan tumor dataset. Breast cancer subtypes were defined by the research version of PAM50 classification [18]. **(B) **Two subsets of embryonic genes from embryonic signature activated in basal-like breast cancers and three subsets found repressed in basal-like breast cancers and activated in luminal tumors and their functional annotation. **(C) **Proliferating cells are observed in the embryonic mammary bud epithelium with Ki67 stain. **(D) **Network of embryonic genes found activated and repressed in basal-like breast cancers in Natrajan data set. **(E) **Box plots showing the average expression levels of the 37 embryonic genes in the breast cancer subtypes classified by using PAM50 SSP on the NKI295 dataset. **(F) **Kaplan-Meier analysis shows significantly reduced distant metastasis-free survival in patients with tumors with activation of embryonic mammary signature in the van de Vijver dataset [15] (χ^2 ^*P *value = 0.0028; log-rank *P *value = 0.0044).

First, we compared the embryonic mammary epithelial signature with those of human breast cancers by using expression arrays from a dataset of 48 grade III ductal carcinomas that were microdissected so that at least 90% of the sample contained tumor cells [16]. The embryonic and tumor datasets profiled microdissected tissues and reflected gene signatures present in highly purified epithelial cell populations isolated from intact tissues. One cluster of 30 embryonic mammary epithelial genes, enriched for regulators of transcription and actin cytoskeleton organization (see Additional File 4), was found to be activated predominantly in ER-negative breast cancers, including all 13 basal-like tumors, all five HER2-positive tumors, and four (13%) of 30 Luminal B tumors (Figure 3A, B). Another small basal-like tumor-associated subset was composed of genes encoding cell-cycle and microtubule cytoskeleton components, suggesting significant overlap with proliferation signatures, a general hallmark of poor-prognosis breast cancers [32] (see Additional file 4). The embryonic mammary epithelium displays a relatively low proliferation index at E12.5, but Ki67^+ ^epithelial cells can be detected at this stage (Figure 3C). Three other subsets of the embryonic mammary epithelial signature are activated in many non-basal-like tumor types (Figure 3B). One cluster activated predominantly in luminal tumors and repressed in most basal-like tumors consists of genes regulating neuron-projection development (Additional file 4). Two other clusters are activated in some luminal and HER2^+ ^tumors and are enriched for genes involved in embryonic appendage morphogenesis, ossification, regionalization, negative regulation of macromolecule synthesis, and wound response (Additional file 4). The stability of the gene clusters was assessed with pvClust (see Additional file 5). Of 57 genes activated in basal-like breast cancers, 55 are found in one of the two major clusters, which have robustness indices larger than 95%. Network analysis suggests complex genetic regulatory potential, and interacting associations exist between the proteins encoded by embryonic genes found activated and repressed in breast cancers (Figure 3D).

We also compared the embryonic mammary epithelial signature with two additional breast cancer datasets, the UNC337 dataset [17,33] and NKI295 dataset [15]. Distinct subsets of the embryonic mammary epithelial signature were shown to be activated in breast cancers; many were similar to those observed in the Natrajan dataset (see Additional files 6, 7, and 8). Five genes are found activated in the mouse *Brca1*^-/- ^tumor dataset and the three breast cancer datasets, predominantly in basal-like cancers; statistical analysis indicated significant enrichment of these genes (see Additional file 9). These included two transcription factors, *Bcl11a *and *Sox11*, and three other genes: *B3gnt5, Ptdss1*, and *Tpx2*. Fifty-seven genes activated in at least two of four tumor datasets displayed enrichment of cell-cycle components (Additional file 3). When 18 proliferation/cell-cycle-associated genes (from signatures described [21]) were removed, 39 remaining genes showed enrichment for embryonic morphogenesis, suggesting that tumor-associated genes mediate proliferation and processes associated with embryonic development in basal-like cancers (Additional file 9). Fifty genes found activated predominantly in non-basal-like types of breast cancers were enriched for neuronal projection/differentiation and ossification, suggesting potential links to regulation of cellular processes regulating bone and nerve development in other breast cancer subtypes (Additional file 10).

Many embryonic mammary signature components, including *ASPM, CDCA2, and KIF20A*, are highly correlated with established proliferation genes, such as *KIF23 *(58%) and *TPX2 *(69%) in the Natrajan dataset and with *TOP2A *(39%*), MKI67 *(36%), and Ki67 protein expression (28%) in the Ghazoui *et al*. dataset [23] (Additional file 10). We defined a 37-gene nonproliferative embryonic mammary signature by excluding two genes found present within two additional published proliferation signatures [22,23] (see Additional file 11). When used in hierarchic cluster analysis, this gene list resulted in robust clustering of basal-like and non-basal-like cancers in the Natrajan dataset. In addition, in the UNC337 and NKI295 datasets, stable basal-like clusters were observed (see Additional file 12). Different single-sample predictors (SSPs) were used to classify the breast cancer subtypes in the original publications. Given the differences in the classification of breast cancers into the molecular subtypes by means of SSPs [18,34], we retrieved the research version of the PAM50 classification for the Natrajan dataset [16] and NKI295 dataset [15] from [18] and PAM50 classification of the UNC337 dataset from [17]. Expression levels of the embryonic gene signature were shown to be highest in basal-like breast cancers compared with the other breast cancer subtypes (Figure 3E). Enrichment for the 37-gene nonproliferative embryonic signature was correlated with reduced-distance metastasis-free survival, larger tumor size, and the 70-gene signature used for prognostication of breast cancer patients [15,19] in the NKI295 dataset (Figure 3F; Additional file 13).

Given that many cancer cells undergo some degree of epithelial-mesenchymal transition (EMT), we also defined an embryonic mammary mesenchymal signature based on expression profiles of genes found highly expressed within embryonic mammary mesenchymal tissue compared with postnatal mammary cells (see Additional file 14). We found that a large percentage (62%) of the mesenchymal genes are components of the embryonic mammary epithelial signature, consistent with these epithelial cells undergoing morphogenesis and harboring some inherent mesenchymal-like traits. Of the overlapping mesenchymal genes, 25 were found in the 37-gene tumor-associated embryonic epithelial signature, and could be considered candidate regulators of EMT in breast cancers (see Additional file 15).

We next defined a tumor-associated mesenchymal signature. We used the criterion of genes found to be activated in basal-like cancers of at least two of four datasets, and we removed genes that overlapped with the epithelial signature. The final embryonic mesenchymal signature would represent transcriptomic features unique to the embryonic stroma. Several of these strictly mesenchymal signature components (*TGFBI, TWIST2, ZEB2*) have established links to EMT [35-37]. Enrichment for the 172-gene mammary mesenchymal signature was correlated with large tumor size and the 70-gene prognostic signature [15,19] in the NKI295 dataset (Additional file 15). No significant association with overall survival was observed in patients whose breast cancers showed activation of the embryonic mesenchymal signature (see Additional file 16).

**Figure 4:**
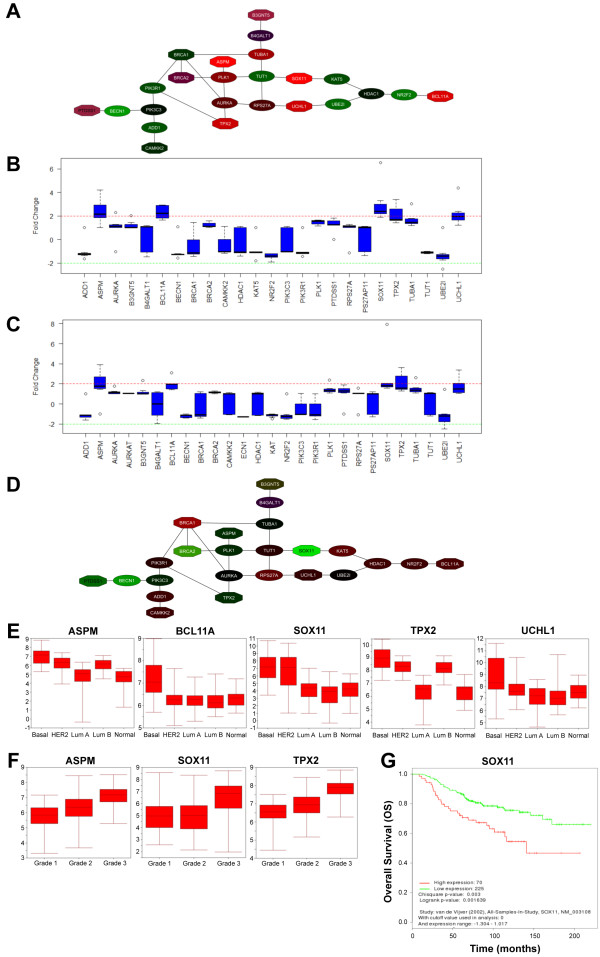
**Core tumor-associated embryonic mammary genes associate significantly with key clinical parameters in breast cancers**. **(A) **Expression levels of core network activated across independent tumor datasets in ER^+ ^versus ER^- ^breast cancers. Red indicates expression levels upregulated in ER^- ^versus ER^+ ^tumors; green indicates expression levels up in ER^+ ^versus ER^- ^tumors. **(B) **Five genes (*ASPM, BCL11A, SOX11, TPX2*, and *UCHL1*) from the core network in Figure 5A show at least a twofold increase in expression levels in ER^- ^versus ER^+ ^breast cancers in seven datasets [16,38-43]. **(C) **Five genes (*ASPM, BCL11A, SOX11, TPX2*, and *UCHL1*) from the core network shown in Figure 5A show at least a twofold increase in expression levels in PR^- ^versus PR^+ ^breast cancers in eight datasets [16,39,41-46]. **(D) **Expression levels of core network activated across six independent tumor datasets [16,40,42,47-49] in HER2^- ^versus HER^+ ^breast cancers. Red, expression levels upregulated in HER2^- ^versus HER2^+ ^tumors; green, expression levels upregulated in HER2^+ ^versus HER2^- ^tumors. **(E) **Significance analysis of microarray (SAM) analysis of *ASPM, BCL11A, SOX11, UCHL1*, and *TPX2 *expression according to tumor subtype, as defined by PAM50 [17], in breast cancers in the Lu dataset [40]. **(F) **SAM analysis of *ASPM, SOX11*, and *TPX2 *expression according to grade in breast cancers in Miller dataset [41]. **(G) **Kaplan-Meier analysis shows significantly reduced overall survival in the high *SOX11 *as compared with the low-*SOX11 *subgroup in the van de Vijver dataset [15] (χ^2 ^*P *value = 0.004; log-rank *P *value = 0.002133).

**Figure 5:**
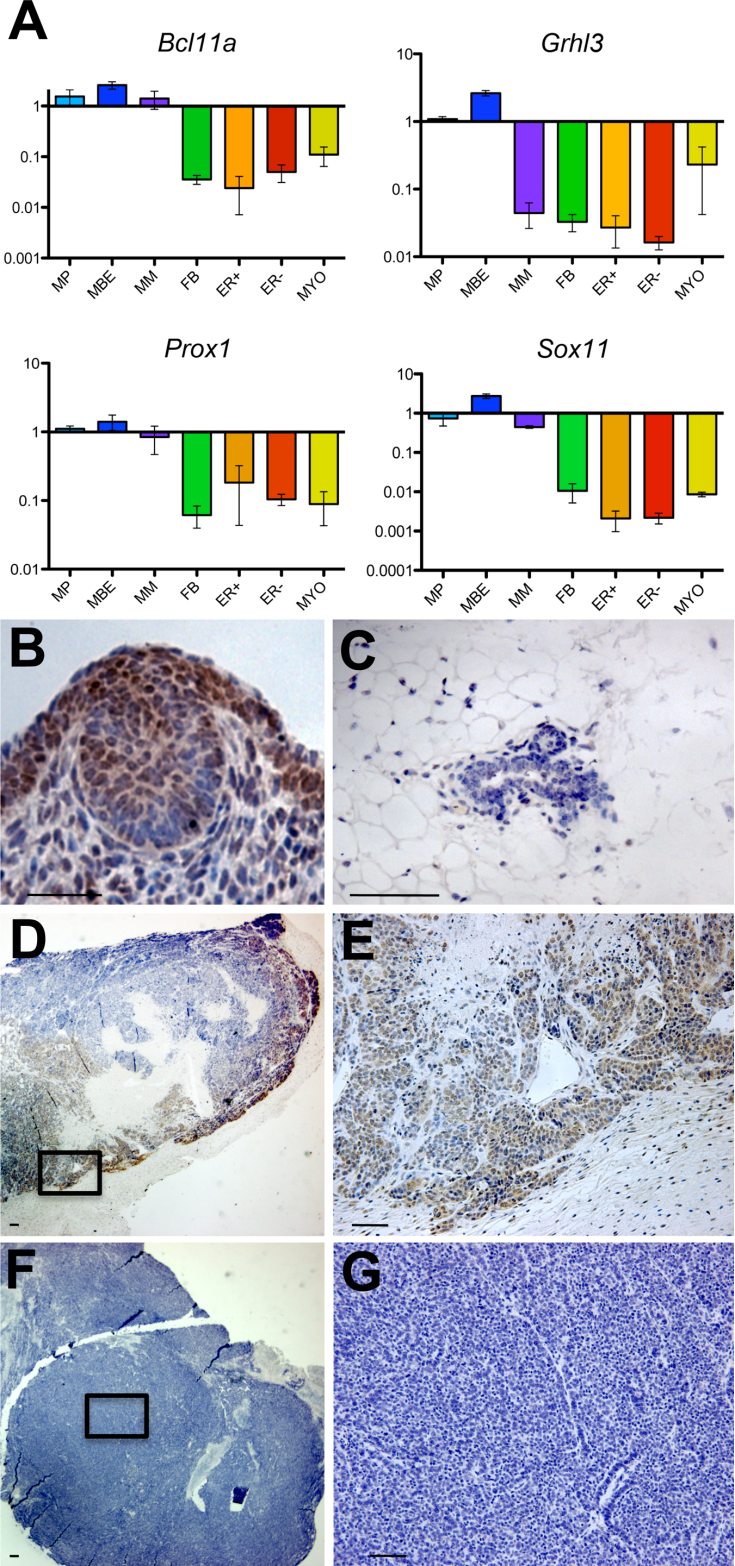
**Embryonic mammary transcription factors are highly expressed in some *Brca1^-/- ^*mammary tumor cells**. **(A) **qRT-PCR data confirming embryonic-enriched expression of several basal-like tumor-associated transcription factors when compared with postnatal MEC subpopulations (described in [10] and [13]). MP, mammary primordium; MBE, E12.5-stage mammary bud epithelium; MM, E12.5-stage mammary mesenchyme; FB, fibroblast; ER^-^, luminal estrogen receptor negative; ER^+^, luminal estrogen-receptor positive; MYO, myoepithelial. **(B) **IHC showing cell types expressing embryonic mammary marker, Sox11 (guinea-pig antiserum) within embryonic mammary primordium. **(C) **IHC showing low level of Sox11 expression (guinea-pig antiserum) within 10-week-old postnatal mammary gland. **(D-G) **IHC showing Sox11 expression (guinea-pig antiserum) in some, but not all, *Brca1^-/- ^*tumors. Scale bar, 50 μm.

*BCL11A, SOX11*, and *TPX2 *showed consistent upregulation at an average of twofold or greater in ER^- ^breast cancers across datasets (Figure 4A, B; Additional file 17) [16,38-43]. *SOX11 *and *TPX2 *showed consistent upregulation of twofold or greater in PR^- ^breast cancers across datasets (Figure 4C; Additional file 17) [16,39,41-46]. *SOX11 *levels were consistently twofold higher in HER2^+ ^versus HER2^- ^samples across datasets [16,40,42,47-49] (Figure 4D and Additional file 18). *SOX11 *levels were higher in basal-like and HER2^+ ^breast cancers compared with other subtypes (Figure 5E). *BCL11A *levels were consistently higher in basal-like breast cancers compared with other subtypes (Figure 4E). Both *SOX11 *and *TPX2 *showed a trend of increased expression levels with increasing tumor grade, whereas *BCL11A *did not (Figure 4F; Additional file 19). *B3GNT5 *levels tended to be higher in both ER-negative and PR-negative tumors. No significant association of *PTDSS1 *with ER^- ^, PR^-^, HER2^- ^status, or histologic grade was found.

Several of the 52 genes found highly expressed in at least two tumor datasets showed consistent trends in expression within tumor subtypes. *UCHL1 *is generally found expressed at higher levels in basal-like tumors than the other breast cancer subtypes (Figure 4E). Many cell-cycle-associated genes (*ASPM, CENPE, FAM60A, TPX2, TRIP13, KIF11, KIF20A*) were expressed at the highest levels in basal-like tumors followed by HER2^+^, LumB, Normal, and LumA (Figure 4E). Similar trends for the cell-cycle-associated genes (*ASPM, CENPE, TPX2, TRIP13, KIF11, KIF20A*) were observed with their distribution in different-grade tumors, with higher expression levels observed as tumor grade increased (Figure 4F). Patients with breast cancers expressing higher levels of *SOX11 *showed worse overall survival than did those with tumors expressing lower levels (Figure 4G). A trend exists for reduced distant metastasis-free survival in patients with breast cancers expressing higher levels of *SOX11*, but is not statistically significant.

### Tumor-associated embryonic mammary transcriptional regulators are expressed in invasive *Brca1^-/- ^*mammary tumor cells

We analyzed expression of four embryonic mammary signature components that encode transcription factors in normal mammary tissues and tumors. *Bcl11a *and *Sox11 *were expressed at approximately 20-fold and 100-fold greater levels, respectively, in the embryonic mammary epithelium when compared with postnatal mammary epithelial cell (MEC) populations when assayed with qRT-PCR (Figure 5A). Expression was also detected in RNA isolated from *Brca1^-/- ^*mouse mammary tumors: *Bcl11a *was detected in seven of eight tumors, and *Sox11 *was detected in two of eight tumors (see Additional file 20). *Grhl3 *and *Prox1 *were expressed at 10-fold or more in the embryonic mammary epithelium when compared with postnatal MEC expression levels (Figure 5A) and were expressed in some *Brca1*^-/- ^tumors when profiled by qRT-PCR (Additional file 20). Sox11 expression is predominantly observed in epidermal cells of the E12.5-stage mammary bud (Figure 5B). Weak expression of Sox11 is detected in postnatal MECs (Figure 5C). Nuclear Sox11 expression is observed in two of eight *Brca1^-/- ^*tumors, with highest levels of expression observed at the tumor-invasion front adjacent to normal tissue (Figure 5D through 5G). We conclude that several signature components identified in our cancer dataset analysis are highly embryonic enriched, expressed at sites of active tissue remodeling *in vivo *during embryonic mammary development and in many *Brca1^-/- ^*mammary tumors.

### *SOX11 *knockdown and overexpression in breast cancer cells

**Figure 6:**
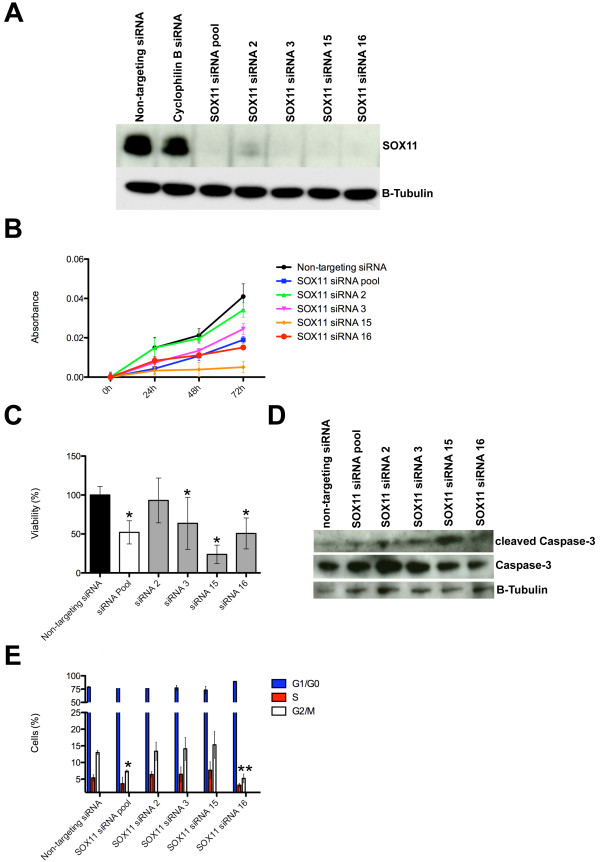
**Effects of *SOX11 *knockdown on cell proliferation and viability of breast cancer cells**. **(A) **SOX11 expression levels in BT474 cells transfected with either *SOX11 *or control siRNAs. SOX11 was detected with immunoblotting. **(B) **BT474 cell number, represented as measured by absorbance by using PrestoBlue cell-viability reagent, after transfection with *SOX11 *or nontargeting siRNAs at daily intervals. Values represent mean ± SEM for three different experiments. **(C) **Change in percentage of viable cells was assessed by using PrestoBlue cell-viability assay of BT474 cells 72 hours after transfection with *SOX11 *siRNAs compared with control siRNA. Values represent mean ± SD for three different experiments; *P < 0.001, compared with the control. **(D) **Caspase-3 and cleaved caspase-3 levels detected by immunoblotting of lysates of adherent and floating BT474 cells transfected with either *SOX11 *or control siRNAs. **(E) **After transfection with either *SOX11 *or control siRNA, adherent BT474 cells were collected and stained with 7AAD. Cell-cycle phase was determined with FACS analysis. Values represent mean ± SD for four independent experiments. *P < 0.05; and **P < 0.01 compared with the control.

We carried out loss-of-function assays to study further the role of SOX11 in BT474 and BT549 invasive breast cancer cells, which express relatively high (BT474) and low levels (BT549) of SOX11 (see Additional file 21). The results indicated that *SOX11 *knockdown significantly impaired the viability and proliferation of both cell types (Figure 6A-C and Additional file 21). BT549 cells transiently transfected with pCMV6-AC-SOX11-GFP exhibited higher proliferation rates than did BT549 cells transiently transfected with a control GFP-expressing plasmid (Additional file 21). *SOX11 *knockdown in BT474 cells increased levels of cleaved caspase-3, a marker for apoptosis (Figure 6D). A significant reduction in cells in G_2_/M phase was observed in cells transfected with both the *SOX11 *SMARTpool and siRNA16, but not with the siRNA15, which exhibits the largest change in cell viability and largest increase in cleaved caspase-3 levels on *SOX11 *knockdown (Figure 6B through 6E).

## Discussion

Embryonic mammary epithelium represents the least differentiated mammary cells. Tumor-associated embryonic mammary epithelial gene activation may therefore reflect tumors containing a large proportion of less-differentiated cells. Differentiation status, as defined by histologic grade, is a clinically relevant aspect of breast tumors [50]. Undifferentiated tumors generally have a much worse prognosis than do more-differentiated tumors [50]. A small component of the embryonic-specific mammary signature appears activated in mouse *Brca1^-/- ^*tumors and in approximately 80% of human basal-like breast cancers in the datasets we examined. It is unclear whether they express these programs for the same reasons or if their expression in basal-like/triple-negative breast cancers is due to genetic aberrations they harbor.

Many of the most common breast cancer driver mutations, which confer survival advantage to breast cancer cells and are implicated in causing cancers to form, are found in genes that are also highly expressed by prenatal breast cells. We have established this by comparing gene-expression profiles of embryonic mammary tissues [1] with recent mutational analyses obtained through deep sequencing of breast cancers [2]. Aspects of embryonic genetic programs with relevance to cancer have also been suggested because "embryonic stem cell-like" (ESC) signatures are found activated in many cancers, including aggressive breast cancers [21]. However, most of these signatures show a very strong correlation with levels of proliferation-related genes [32,51,52]. Although we observe correlation with proliferation for embryonic mammary signature components after removing proliferation-associated genes, we still observed clustering into basal-like and non-basal breast cancers, suggesting that the embryonic gene activation is also mediating other cellular processes.

Four transcription factors (*Bcl11a, Grhl3, Prox1, Sox11*) activated in *Brca1*^-/- ^mouse tumors and basal-like human breast cancers across multiple datasets were chosen for validation studies, and all were confirmed to be embryonic-enriched and highly expressed by some tumors. All four genes have links to progenitor-cell regulation. *GRHL3 *collaborates with Trithorax group members to activate the epidermal progenitor differentiation program [53]. Prox1 has been identified as a suppressor of hematopoietic stem cell activity [54], primary mediator of lymphangiogenesis [55], and promotes maintenance of intermediate neural progenitors during adult neurogenesis [56]. *BCL11A *is expressed in lymphohematopoietic cells, controls the development of B- and T-lymphocytes, and is a common site of retroviral integration in myeloid leukemia [57,58]. Two somatic mutations in *BCL11A *have been reported in breast cancer [59]. *Sox11*, a high-mobility-group transcription factor, has a widespread role for in tissue remodeling in multiple organs [60] and regulates neurogenesis [61,62]. Activated *SOX11 *expression has been described in Wilms tumor [63], a classic example of an embryonic tumor, often characterized by retention of embryonic cellular structures within the tumor-bearing kidney [63]. SOX11 plays pivotal roles in lymphoblastic neoplasms, mantle cell lymphoma, and Burkitt lymphoma [64]. Both *BCL11A *and *SOX11 *belong to the top 20 transcriptional regulators that correlate with the core ES signature found activated in aggressive breast cancers [21].

Antibody staining found Sox11 highly expressed at the invasion front in some *Brca1^-/- ^*mammary tumors. *SOX11 *has been identified as mesenchymal stem cell (MSC) characteristic gene [65] and potential biomarker for early progenitor human MSCs [66]. Knockdown of *SOX11 *suppressed the self-renewal capacity and differentiation potential of multiple MSC lines [65] and MSCs isolated from bone marrow aspirates [66]. In mice, *Sox11 *is required for proliferation of the sympathetic ganglia during early developmental stages [28]. We found that silencing of *SOX11 *in breast cancer cells led to an increased expression of the apoptotic marker, cleaved caspase-3. *SOX11*-deficient cell populations that showed moderate decreases in viability also exhibited moderate increases in cleaved caspase-3 levels and decreased percentages of cells in G_2_/M phase, whereas no change in the G_2_/M percentage was observed in the least viable *SOX11*-deficient cell population that displayed the highest increase in cleaved caspase-3 levels. These results suggest that a more efficient *SOX11 *knockdown could lead to more rapid apoptosis when compared with cells with moderately reduced *SOX11 *levels, which may possibly undergo prolonged cell-cycle arrest before subsequent apoptosis. A number of studies have found that *Sox11 *is also required for survival of neural cells and mesenchymal progenitor cells [67-69]. We found that silencing of *SOX11 *in breast cancer cells reduces cell survival and cell viability, and SOX11 overexpression leads to increased proliferation rates, suggesting that SOX11 could have a similar function in regulation of proliferation and survival in several types of cells. High levels of *SOX11 *expression are associated with poor overall survival in breast cancer patients, but its function in breast epithelial cells is not clear and remains to be further investigated.

A study by Spike *et al. *[30], found evidence of molecular similarity of subpopulations of E18.5-stage mammary cells to breast cancers. In that study [30], cells from mammary primordia were separated into subpopulations based on expression of cell-surface markers to enrich for stem cells. The signature used in our study represents the entire embryonic mammary epithelial organogenetic program, because it is derived from gene-expression profiles of intact epithelial tissues. Lineage-tracing studies have shown that embryonic mammary bud epithelial cells labeled at midgestation (E12.5-stage) onward give rise to both basal and luminal lineages [70,71]. Therefore, our embryonic signature will include progenitor/stem cells as well as other cells within their native microenvironment. Tumors are composed of multiple cell types, and some behavioral features are similar to organotypic growth [72]. Distinctions in both the developmental stages (E12.5 versus E18.5 stage) and biologic features of the cell populations (tissues versus fractionated subpopulations of dissociated cells) that were profiled in the two studies are likely to account for the limited overlap between the signatures. Only one gene (*Bcl11a*) from the 37-gene signature defined here is shared with one of the tumor-associated subsets defined in the study by Spike *et al. *[30].

Our results reveal a small number of genes associated with embryonic mammary development and human basal-like breast cancers. Although this lends support to the notion that reactivation of components of the mammary organogenetic program has detrimental effects in postnatal MECs, our results suggest that only a small fraction of the early (E12.5-stage) embryonic mammary developmental program is highly expressed or reactivated in breast tumors. A substantial component of the tumor-associated embryonic epithelial signature comprises genes regulating cell proliferation. This is somewhat unexpected, because the embryonic mammary epithelium exhibits a low proliferation index [73,74]. However, several cell-cycle-associated genes are associated with its signature, and may regulate proliferation of particular progenitor cells as the immature mammary cell population expands. One tumor-associated embryonic gene, *ASPM*, regulates symmetric versus asymmetric cell divisions in progenitor cells and also regulates WNT signaling in the developing brain [75,76].

We documented activation of embryonic genes in mammary tumors from mice in which *Brca1^-/- ^*was inactivated in either luminal progenitor cells or basal cells [12]. These observations suggest that it is loss of *Brca1 *and not the cell of origin that may be dictating the embryonic gene signature expression. These *Brca1^-/- ^*mice were *Tp53^+/-^*; hence, it is possible that loss of p53 function is also contributing to the observed embryonic gene activation in the *Brca1^-/- ^*tumors. p53 has been shown to regulate polarity of cell division in mammary stem cells, and loss of p53 appears to promote symmetric divisions of cancer stem cells, contributing to tumor growth [77].

## Conclusions

A limited subset of the early mammary developmental program is likely to have a role in promoting tumorigenesis, but its association with some human breast tumors and patient outcome warrants further investigation. We have identified a small network of embryonic genes that are found highly expressed in a subset of basal-like breast cancers and are candidate regulators of cancer cells. These results provide support for the notion that overactivation of small particular aspects of the embryonic mammary genetic program could play a key role in regulating detrimental cellular behaviour, such as tissue remodeling, invasive growth, and/or progenitor cell expansion. Expression of particular embryonic mammary markers within tumor cells may reflect reactivation of genetic programs that influence the behavior of immature cell types present within the breast and may elicit cell behavior associated with embryonic cells, such as a less-differentiated, highly plastic state. Tumor-associated embryonic mammary markers may have value to be exploited as they could represent a novel means to describe and categorize the biologic state of tumor cell populations for use in breast cancer classification as well as potential drug targets.

## Abbreviations

ER: estrogen receptor; ESC: embryonic stem cell; Krt: keratin; MBE: mammary bud epithelium; MEC: mammary epithelial cell; MSC: mesenchymal stem cell; PR: progesterone receptor; SSPs: single-sample predictors.

## Competing interests

The authors declare that they have no competing interests.

## Authors' contributions

BAH conceived of and designed the study and wrote the manuscript. MZ, QG, and AM carried out analyses. MJS and JSR-F provided guidance and samples and participated in the preparation of the manuscript. EO, OW, and HK performed the experimental work. All authors read and approved the manuscript for publication.

## Supplementary Material

Additional file 1 **Antibodies used for immunohistochemistry and whole-mount immunofluorescence**. **(A) **Table gives details of antibodies used in this study. (**B) **Positive control for Sox11 (guinea-pig antiserum) staining of E12.5-stage forebrain. **(C) **No primary antibody control for Sox11 (guinea-pig antiserum) staining of E12.5-stage mammary primordium. Scale bar, 50 μm. Click here for file

Additional file 2 **Embryonic mammary epithelial signature and functional annotation clustering**. Table of embryonic mammary epithelial signature based on the expression profiles of genes found highly expressed (10-fold or greater) within mammary bud epithelial cells when compared with postnatal mammary epithelial cells and functional annotation clustering of the embryonic mammary epithelial gene signature. Genes shared by this signature and the "uniquely fetal mammary stem cell" signature defined by Spike *et al. *[30] are indicated. Click here for file

Additional file 3 **Embryonic genes found activated in mouse *Brca1^-/- ^*tumors**. Table shows embryonic genes found activated in mouse *Brca1^-/- ^*tumors and functional-annotation clustering. Functional-analysis clustering lists the category of gene set (for example, CC, cellular location; BP, biologic process; MF, molecular function); term (that is, specific gene ontology (GO) with GO number); count (number of genes enriching term); % (percentage of total of genes that belong to category enriched by analyzed gene set); *P *value (that is, enrichment of gene set); genes (list of genes enriching gene set by Affymetrix ID); Bonferroni; Benjamini, and FDR (false discovery rate) for functional annotation clustering of genes expressed in tumor-associated gene modules defined by cluster analysis. Click here for file

Additional file 4 **Embryonic genes found activated and repressed in basal-like, HER2^+^, or luminal breast cancer subtypes in Natrajan data set**. Functional-analysis clustering lists the category of gene set (CC, cellular location; BP, biologic process; MF, molecular function); term (specific gene ontology (GO) with GO number); count (number of genes enriching term); % (percentage of total of genes that belong to category enriched by analyzed gene set); *P *value (enrichment of gene set); genes (list of genes enriching gene set by Affymetrix ID); Bonferroni; Benjamini, and FDR (false discovery rate) for functional-annotation clustering of genes expressed in tumor-associated gene modules defined by cluster analysis. Click here for file

Additional file 5 **Cluster-stability analysis of the hierarchic clustering of the embryonic mammary signature in breast cancer datasets by using the R-package pvclust**. Figure shows stability analysis with Approximately Unbiased (AU) *P *value (shown in green) larger than 95% highlighted by rectangles and strongly supported by data. **(A) **Cluster-stability analysis of the hierarchic clustering of the embryonic mammary signature in the Natrajan breast cancer samples. Of the 57 basal-like genes, 55 are in the left cluster, and the two major clusters are significantly different. **(B) **Cluster-stability analysis of the hierarchic clustering of the embryonic mammary signature in the UNC337 breast cancer samples. **(C) **Cluster-stability analysis of the hierarchic clustering of the embryonic mammary signature in the NKI295 breast cancer samples. Click here for file

Additional file 6 **Similar embryonic epithelial mammary signature subsets are activated across multiple human breast cancer datasets**. **(A, B) **Five embryonic gene clusters activated in UNC337 dataset by using unsupervised hierarchic clustering and functional annotation. Tumor subtypes were defined by PAM50, as described [17]. **(C, D) **Four embryonic gene clusters activated in NKI295 dataset by using unsupervised hierarchic clustering and functional annotation. Subtypes were as defined by the research version of PAM50 classification [18]. The 70-gene prognosis signature was used to classify tumors as to whether tumors are likely to predictive of a short interval to distant metastases (poor) or not (good) [15,19]. Click here for file

Additional file 7 **Embryonic genes found activated and repressed in basal-like, HER2^+^, luminal or normal breast cancer subtypes in UNC337 data set**. Functional-analysis clustering lists the category of gene set (CC, cellular location; BP, biologic process; MF, molecular function); term (specific gene ontology (GO) with GO number); count (number of genes enriching term); % (percentage of total of genes that belong to category enriched by analyzed gene set); *P *value (enrichment of gene set); genes (list of genes enriching gene set by Affymetrix ID); Bonferroni; Benjamini, and FDR (false discovery rate) for functional annotation clustering of genes expressed in tumor-associated gene modules defined by cluster analysis. Click here for file

Additional file 8 **Embryonic genes found activated or repressed in basal-like, HER2^+^, luminal, or normal breast cancer subtypes in NKI295 data set**. Functional-analysis clustering lists the category of gene set (CC, cellular location; BP, biologic process; MF, molecular function); term (specific gene ontology (GO) with GO number); count (number of genes enriching term); % (percentage of total of genes that belong to category enriched by analyzed gene set); *P *value (enrichment of gene set); genes (list of genes enriching gene set by Affymetrix ID); Bonferroni; Benjamini, and FDR (false discovery rate) for functional annotation clustering of genes expressed in tumor-associated gene modules defined by cluster analysis. Click here for file

Additional file 9 **Embryonic genes activated in mouse *Brca1^-/- ^*tumors and basal-like breast cancers, in at least two of four datasets examined and their functional annotation and hypergeometric statistical analysis**. Click here for file

Additional file 10 **Embryonic genes activated in non-basal-like breast cancer subtypes in at least two of three breast cancer datasets examined and their functional annotation**. Click here for file

Additional file 11 **Correlation tests of embryonic genes with proliferation genes used to define nonproliferative embryonic mammary gene signature with proliferation genes, *t *tests of average expression of embryonic genes in tumor subtypes with different SSPs and centroid analysis of nonproliferative gene signature in NKI295 dataset**. **(A) **Pearson correlation of *KIF23 *with embryonic gene signature in Natrajan dataset. **(B) **Pearson correlation of *TPX2 *with embryonic gene signature in Natrajan dataset. **(C) **Pearson correlation of *TOP2A *with embryonic gene signature in FAIMos dataset. **(D) **Pearson correlation of *MKI67 *with embryonic gene signature in FAIMos dataset. **(E) **Spearman correlation of Ki67 protein expression with embryonic gene signature in FAIMos dataset. **(F) **Nonproliferative embryonic mammary gene signature. Click here for file

Additional file 12 **Cluster-stability analysis of the hierarchic clustering of the embryonic mammary signature in breast cancer datasets by using the R-package pvclust**. **(A) **Cluster-stability analysis; 55 of the 57 basal-like genes are in the left cluster, and the two major clusters are significantly different. **(B) **Cluster-stability analysis of the hierarchic clustering of the nonproliferative embryonic mammary signature in the UNC337 breast cancer samples. **(C) **Cluster-stability analysis of the hierarchic clustering of the nonproliferative embryonic mammary signature in the NKI295 breast cancer samples. Click here for file

Additional file 13 **The *t *tests of average expression of embryonic genes in tumor subtypes by using different SSPs and centroid analysis of nonproliferative gene signature**. **(A) **Summary of array-expression data for the 37-gene embryonic mammary epithelial signature used to define centroids. **(B) **Median gene expression of 37 genes comprising embryonic mammary epithelial gene signature. **(C) **Definition of centroids for embryonic mammary epithelial signature. **(D) **Centroid correlation with NKI295 dataset. **(E) **Multivariate Cox Proportional Hazard Regression analysis. Click here for file

Additional file 14 **Embryonic mammary mesenchymal signature and functional annotation clustering**. Table of embryonic mammary epithelial signature based on the expression profiles of genes found highly expressed (10-fold or greater) within mammary mesenchymal cells when compared with postnatal mammary epithelial cells and functional annotation-cluster analysis of the embryonic mammary mesenchymal gene signature. Click here for file

Additional file 15 **Embryonic mesenchymal genes activated in mouse *Brca1^-/- ^*tumors and basal-like breast cancers**. These are from at least two of four datasets examined and Multivariate Cox Proportional Hazard Regression analysis of 172-gene uniquely mesenchymal signature. Click here for file

Additional file 16 **Mesenchymal signature activation in breast cancers**. **(A) **Kaplan-Meier analysis shows a trend toward reduced overall survival in patients with tumors with activation of embryonic mesenchymal signature (172 genes) in the van de Vijver dataset [15] (χ^2 ^*P *value = 0.066, log-rank *P *value = 0.07368). **(B) **Box plots showing the average expression levels of the mesenchymal 172-gene signature in the breast cancer subtypes classified by using PAM50 SSP on the NKI295 dataset. Click here for file

Additional file 17 **Fold-change expression levels of core network components activated across independent tumor datasets**. The average in ER^- ^versus ER^+ ^breast cancers; PR^- ^versus PR^+ ^breast cancers; and HER2^- ^versus HER^+ ^breast cancers, including associated *P *values. Click here for file

Additional file 18 **Expression of core network of tumor-associated embryonic genes in HER2^+ ^versus HER2^- ^breast cancers in six datasets**. Click here for file

Additional file 19 **Significance analysis of microarray analysis of the expression of core network of tumor-associated embryonic genes according to tumor grade and tumor subtype**. Click here for file

Additional file 20 **Expression of embryonic genes in mammary tissues and *Brca1^-/- ^*tumors**. **(A) **qRT-PCR analysis of four tumor-associated transcription factors in *Brca1^-/- ^*mouse mammary tumors. **(B) **IHC showing SOX11 expression (Cell Marque MRQ-58) within embryonic mammary primordium. **(C) **No primary antibody control for SOX11 (Cell Marque MRQ-58). **(D) **IHC showing low level of SOX11 expression (Cell Marque MRQ-58) within 10-week-old postnatal mammary gland. **(E) **Positive control showing SOX11 expression (Cell Marque MRQ-58) in E12.5-stage forebrain. **(F) **Control showing SOX11 expression (Cell Marque MRQ-58) in E16.5-stage *Sox11^-/- ^*spinal cord. **(G **through **J) **IHC showing SOX11 expression (Cell Marque MRQ-58) in some, but not all, *Brca1^-/- ^*tumors. Scale bar, 50 μm. Click here for file

Additional file 21 **Effects of *SOX11 *knockdown on cell viability of breast cancer cells**. ***(*A) **qRT-PCR analysis of *SOX11 *levels in BT549 cells transfected with either *SOX11 *SMARTpool or control siRNAs. (**B) **SOX11 expression in BT474 cells compared with BT549 cells by immunoblotting. **(C) **Immunoblotting of lysates from cells transiently transfected with either SOX4 or SOX11 expression vectors (Origene) show that SOX11 antibody (Epitomics) does not detect SOX4. SOX4 shares a high degree of identity both in the HMG box domain and in the C-terminal region and is of a similar molecular mass to SOX11 (60 versus 59 kDa), in agreement with previously published data [78]. **(D) **BT549 cell number represented as measured by PrestoBlue cell viability reagent after transfection with SOX11 or nontargeting siRNAs at daily intervals. Values represent means ± SD for three different experiments. **(E) **Change in percentage of viable cells was assessed by using PrestoBlue cell-viability assay of BT549 cells 72 hours after transfection with *SOX11 *siRNAs compared with control siRNA. Values represent mean ± SD for three different experiments. *P < 0.05, and ***P < 0.001 compared with the control. **(F)**. Absorbance of BT549 cells transfected with either *SOX11-GFP *or control GFP-expressing plasmid was assessed by using PrestoBlue cell-viability assay at daily intervals. Values represent mean ± SEM for three independent experiments; *P < 0.05, compared with the control. The transfection efficiency was about 24% for the SOX11-GFP-expressing plasmid. Click here for file
