# Supplementary material for: Embryonic mammary signature subsets are activated in Brca1-/- and basal-like breast cancers
Source: Breast Cancer Res. 2013 Mar 18;15(2):R25. doi: 10.1186/bcr3403 (PMC3672751; doi:10.1186/bcr3403)

A. Table of antibodies used in this study.

Antibody	Company	Address	Cat No.	WMIF or IF	Dilution
Ki67	DAKO	Glostrup, Denmark	M7248	WMIF	1:25
Krt5	Covance	Berkley, CA	PRB-160P	WMIF	1:25
CD49f/Itga6	BD Biociences	Franklin Lakes, NJ	555734	WMIF	1.100
Krt14	Covance	Berkley, CA	PRB-159P	WMIF	1.25
p63	Abcam	Cambridge, UK	AB735	IHC / Formalin	1.40
ERalpha	DAKO	Glostrup, Denmark	M7047	IF / Formalin	1.40
PRa7	Neomarkers	Fremont, CA	MS-197-P1	IF / Formalin	1.200
Erbb2	Cell Signalling	Beverley, MA	2165	IF / Formalin	1:10
EGFR	Santa Cruz	Santa Cruz, CA	sc-03	IHC / Formalin	1:50
Sox11	Dr Elisabeth Sock	Institut für Biochemie, Erlangen, Germany		IHC / Formalin	1:500
SOX11	Cell Marque	Rocklin, CA	382M-16	IHC / Formalin	1:50

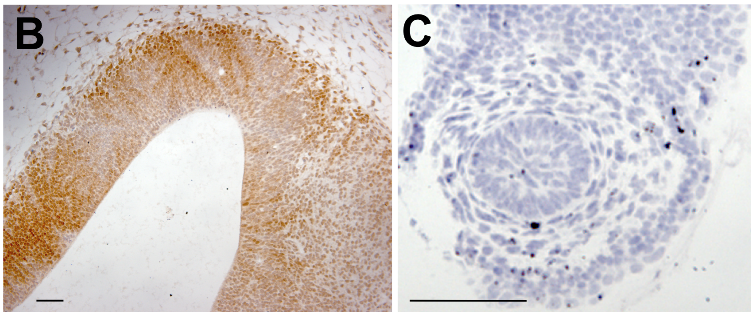

Supplement: Additional file 1 — Antibodies used for immunohistochemistry and whole-mount immunofluorescence. (A) Table gives details of antibodies used in this study. (B) Positive control for Sox11 (guinea-pig antiserum) staining of E12.5-stage forebrain. (C) No primary antibody control for Sox11 (guinea-pig antiserum) staining of E12.5-stage mammary primordium. Scale bar, 50 μm. [file bcr3403-S1.PDF]
