# Supplementary material for: Embryonic mammary signature subsets are activated in Brca1-/- and basal-like breast cancers
Source: Breast Cancer Res. 2013 Mar 18;15(2):R25. doi: 10.1186/bcr3403 (PMC3672751; doi:10.1186/bcr3403)
Supplement: Additional file 5 — Cluster-stability analysis of the hierarchic clustering of the embryonic mammary signature in breast cancer datasets by using the R-package pvclust. Figure shows stability analysis with Approximately Unbiased (AU) P value (shown in green) larger than 95% highlighted by rectangles and strongly supported by data. (A) Cluster-stability analysis of the hierarchic clustering of the embryonic mammary signature in the Natrajan breast cancer samples. Of the 57 basal-like genes, 55 are in the left cluster, and the two major clusters are significantly different. (B) Cluster-stability analysis of the hierarchic clustering of the embryonic mammary signature in the UNC337 breast cancer samples. (C) Cluster-stability analysis of the hierarchic clustering of the embryonic mammary signature in the NKI295 breast cancer samples. [file bcr3403-S5.PDF]

A

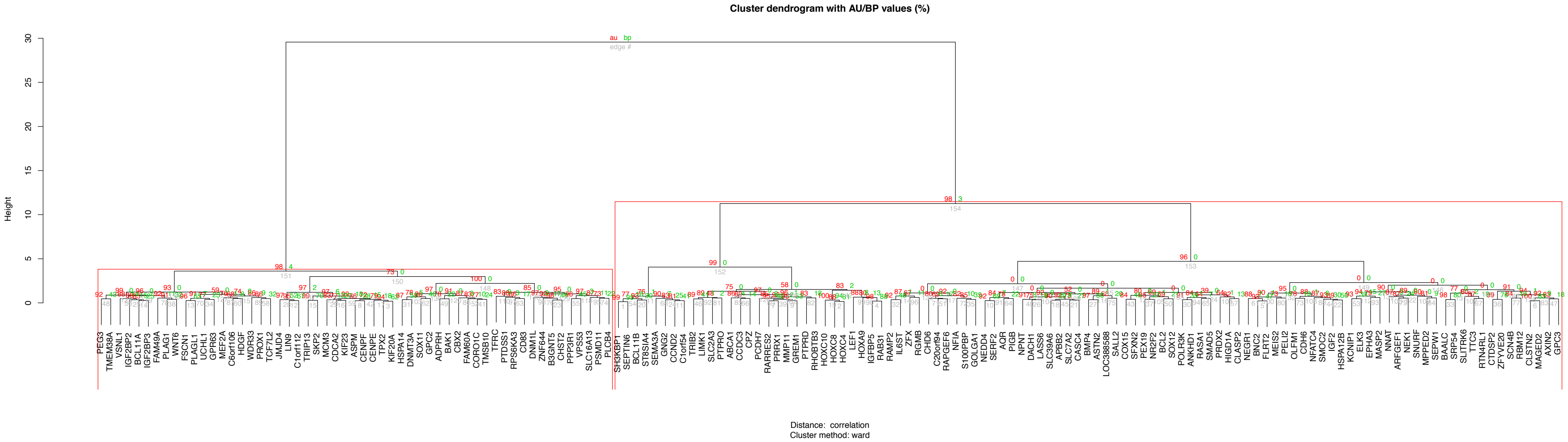

Cluster dendrogram with AU/BP values (%)
au
bp
edge #
Height
30
25
20
15
10
5
0
Distance: correlation
Cluster method: ward

B

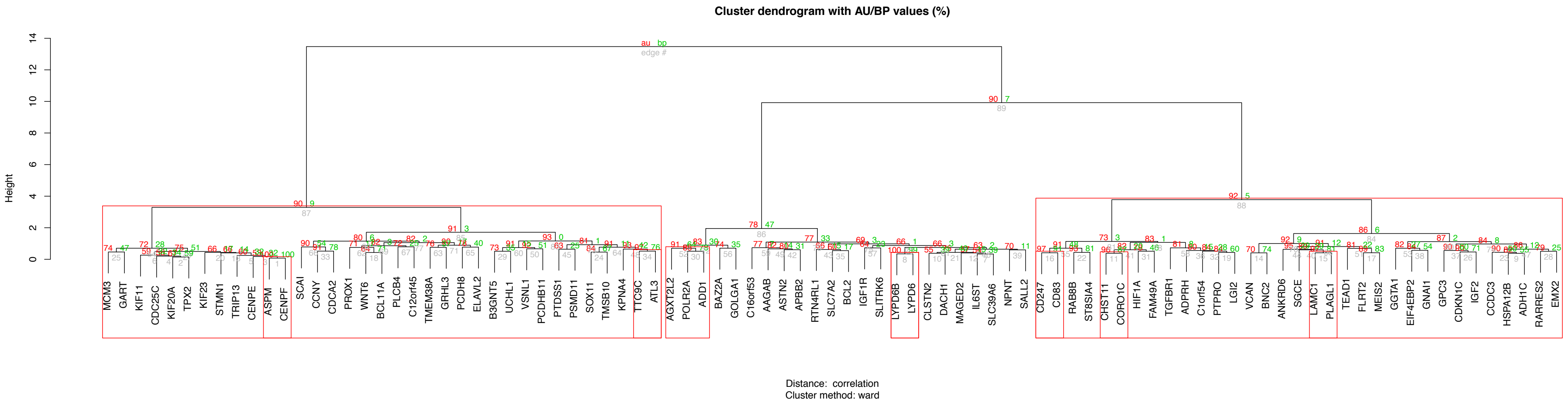

The two major clusters (basal versus non-basal) have robustness indices larger than 90% in the UNC337 dataset.

C

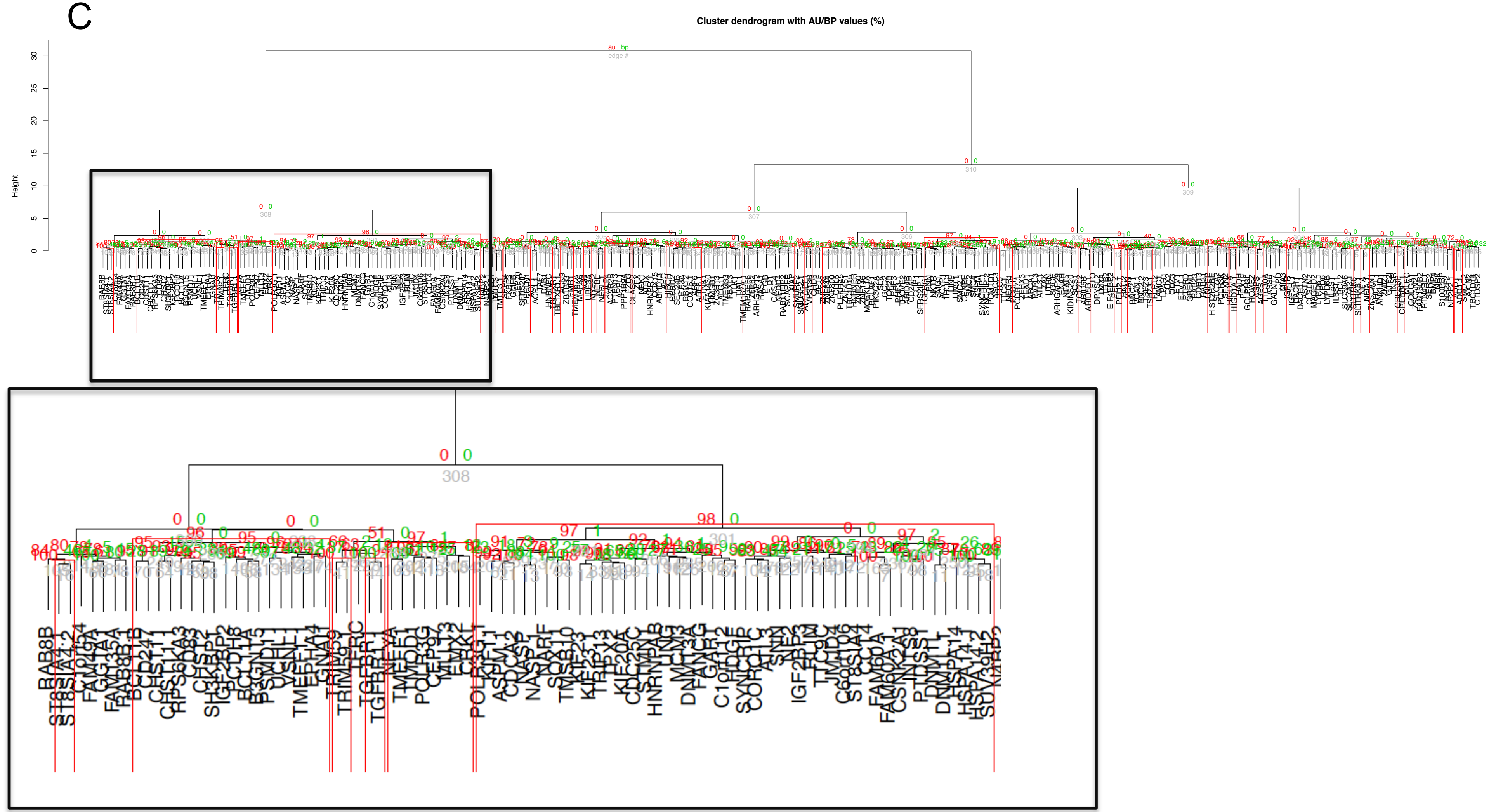

No significant difference between the two major clusters is observed in the NKI295 dataset, although one stable basal cluster is observed when assessed with pvClust.
