# Supplementary figures and images for: Embryonic mammary signature subsets are activated in Brca1-/- and basal-like breast cancers
Source: Breast Cancer Res. 2013 Mar 18;15(2):R25. doi: 10.1186/bcr3403 (PMC3672751; doi:10.1186/bcr3403)

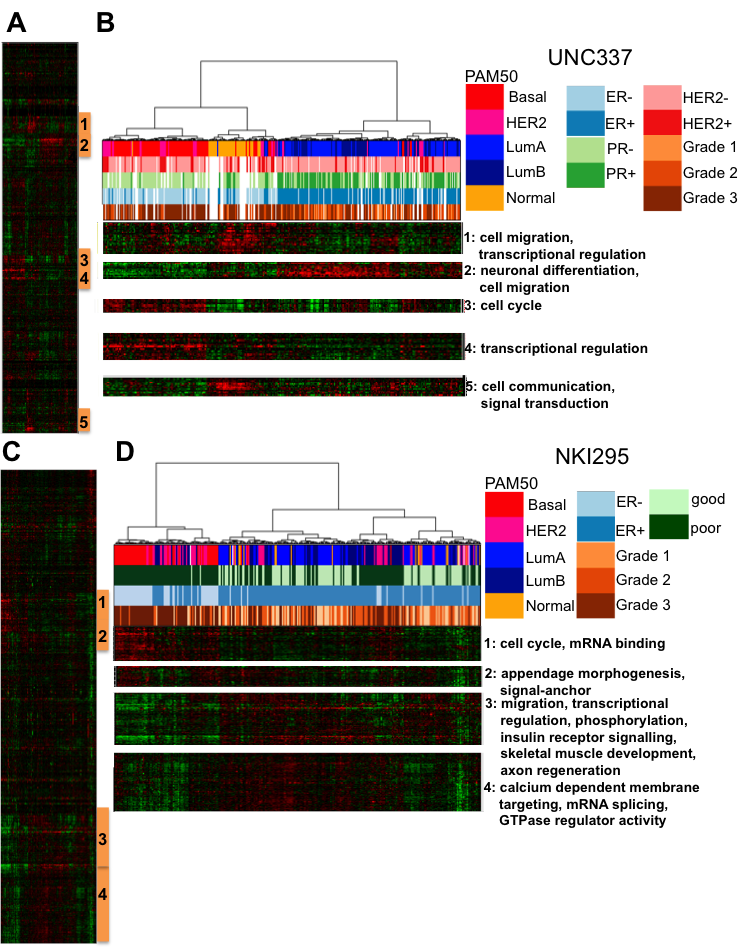

Supplement: Additional file 6 — Similar embryonic epithelial mammary signature subsets are activated across multiple human breast cancer datasets. (A, B) Five embryonic gene clusters activated in UNC337 dataset by using unsupervised hierarchic clustering and functional annotation. Tumor subtypes were defined by PAM50, as described [17]. (C, D) Four embryonic gene clusters activated in NKI295 dataset by using unsupervised hierarchic clustering and functional annotation. Subtypes were as defined by the research version of PAM50 classification [18]. The 70-gene prognosis signature was used to classify tumors as to whether tumors are likely to predictive of a short interval to distant metastases (poor) or not (good) [15,19]. [file bcr3403-S6.TIFF]

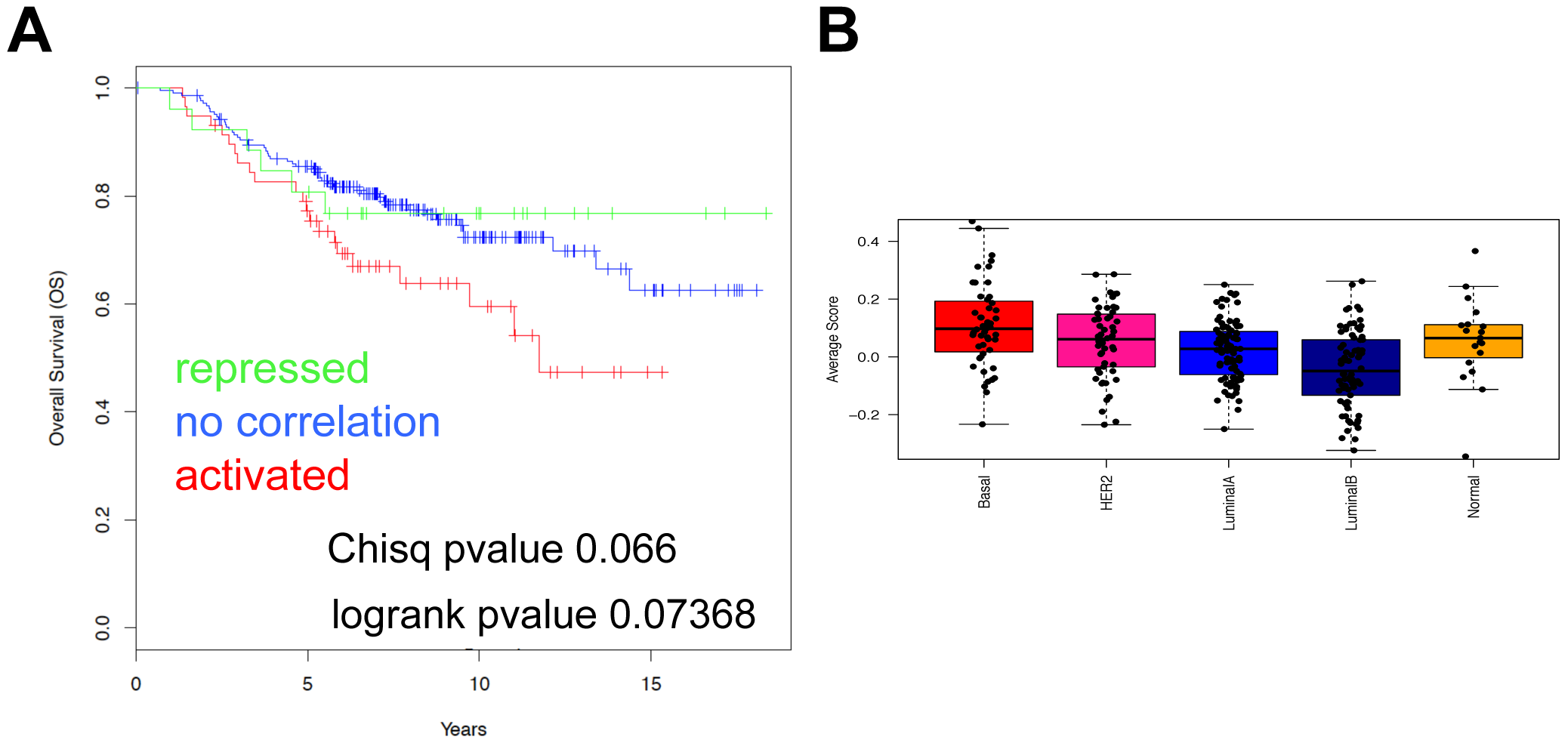

Supplement: Additional file 16 — Mesenchymal signature activation in breast cancers. (A) Kaplan-Meier analysis shows a trend toward reduced overall survival in patients with tumors with activation of embryonic mesenchymal signature (172 genes) in the van de Vijver dataset [15] (χ2 P value = 0.066, log-rank P value = 0.07368). (B) Box plots showing the average expression levels of the mesenchymal 172-gene signature in the breast cancer subtypes classified by using PAM50 SSP on the NKI295 dataset. [file bcr3403-S16.TIFF]

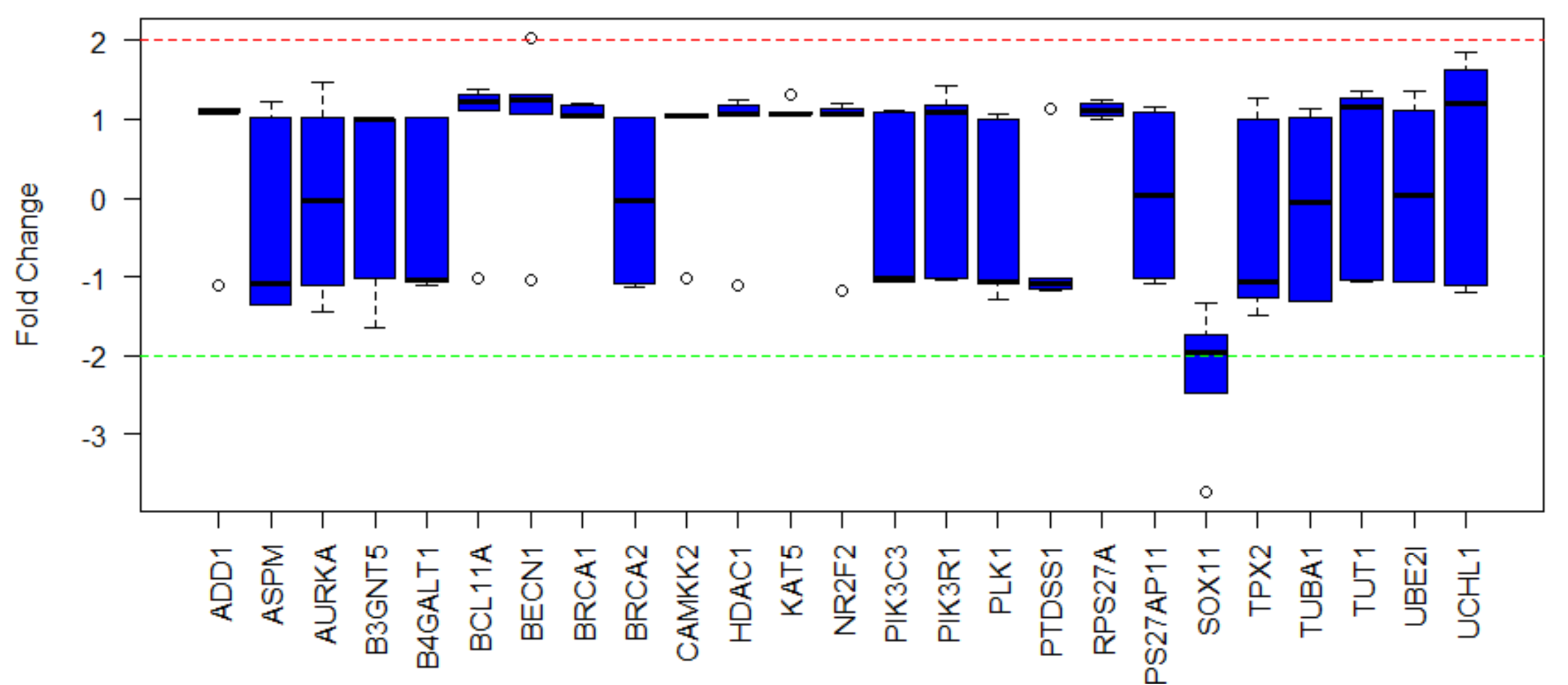

Supplement: Additional file 18 — Expression of core network of tumor-associated embryonic genes in HER2+ versus HER2- breast cancers in six datasets. [file bcr3403-S18.TIFF]

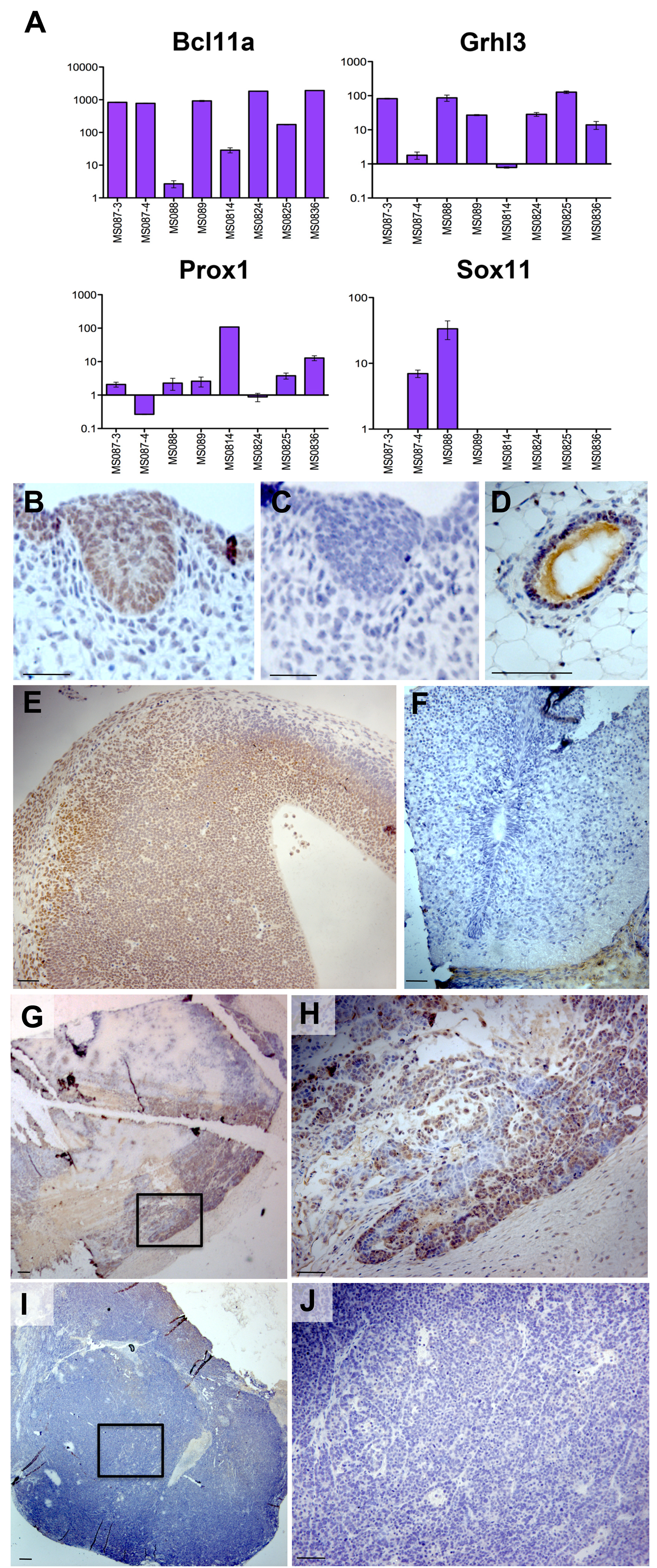

Supplement: Additional file 20 — Expression of embryonic genes in mammary tissues and Brca1-/- tumors. (A) qRT-PCR analysis of four tumor-associated transcription factors in Brca1-/- mouse mammary tumors. (B) IHC showing SOX11 expression (Cell Marque MRQ-58) within embryonic mammary primordium. (C) No primary antibody control for SOX11 (Cell Marque MRQ-58). (D) IHC showing low level of SOX11 expression (Cell Marque MRQ-58) within 10-week-old postnatal mammary gland. (E) Positive control showing SOX11 expression (Cell Marque MRQ-58) in E12.5-stage forebrain. (F) Control showing SOX11 expression (Cell Marque MRQ-58) in E16.5-stage Sox11-/- spinal cord. (G through J) IHC showing SOX11 expression (Cell Marque MRQ-58) in some, but not all, Brca1-/- tumors. Scale bar, 50 μm. [file bcr3403-S20.JPEG]

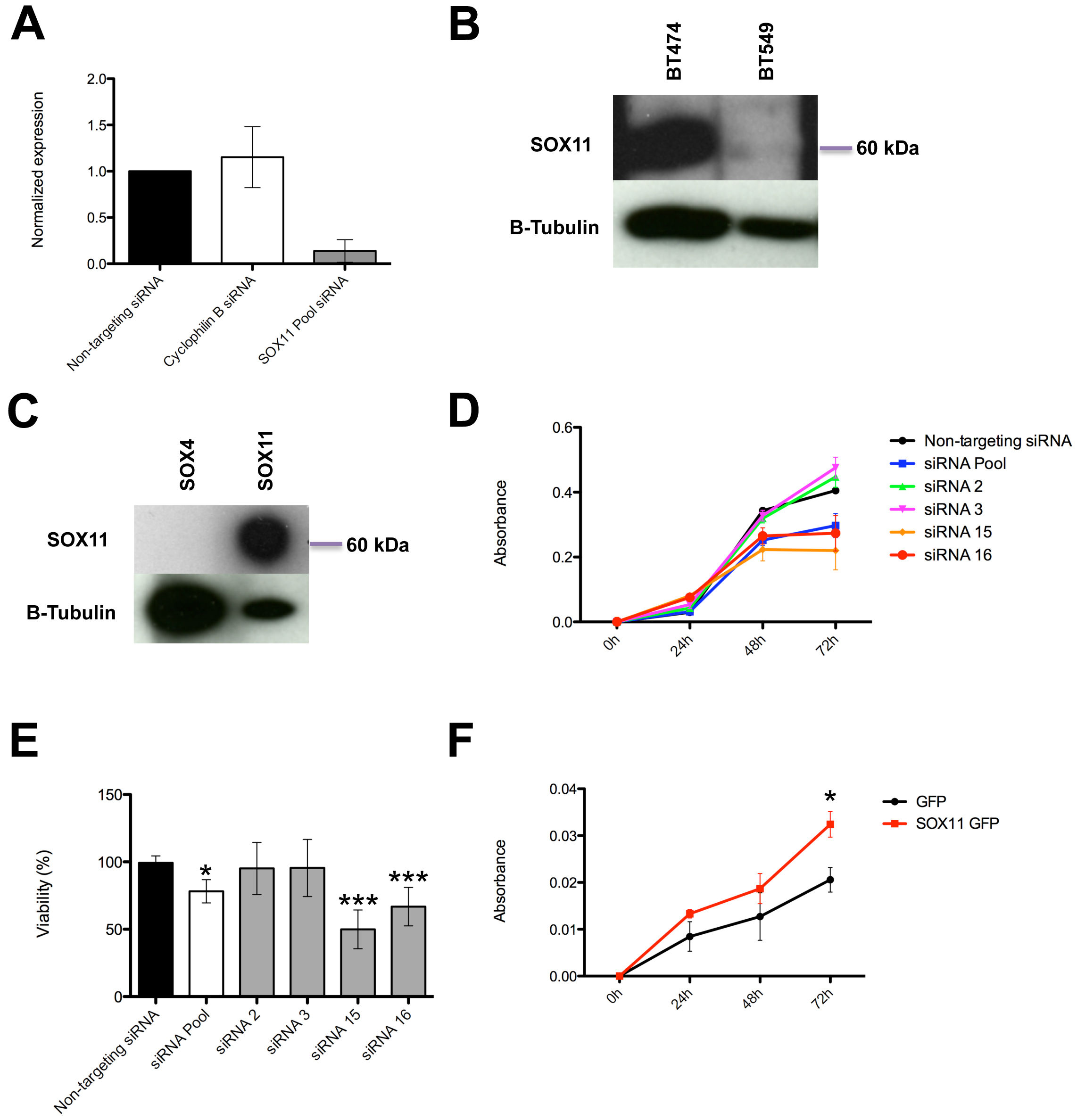

Supplement: Additional file 21 — Effects of SOX11 knockdown on cell viability of breast cancer cells. (A) qRT-PCR analysis of SOX11 levels in BT549 cells transfected with either SOX11 SMARTpool or control siRNAs. (B) SOX11 expression in BT474 cells compared with BT549 cells by immunoblotting. (C) Immunoblotting of lysates from cells transiently transfected with either SOX4 or SOX11 expression vectors (Origene) show that SOX11 antibody (Epitomics) does not detect SOX4. SOX4 shares a high degree of identity both in the HMG box domain and in the C-terminal region and is of a similar molecular mass to SOX11 (60 versus 59 kDa), in agreement with previously published data [78]. (D) BT549 cell number represented as measured by PrestoBlue cell viability reagent after transfection with SOX11 or nontargeting siRNAs at daily intervals. Values represent means ± SD for three different experiments. (E) Change in percentage of viable cells was assessed by using PrestoBlue cell-viability assay of BT549 cells 72 hours after transfection with SOX11 siRNAs compared with control siRNA. Values represent mean ± SD for three different experiments. *P < 0.05, and ***P < 0.001 compared with the control. (F). Absorbance of BT549 cells transfected with either SOX11-GFP or control GFP-expressing plasmid was assessed by using PrestoBlue cell-viability assay at daily intervals. Values represent mean ± SEM for three independent experiments; *P < 0.05, compared with the control. The transfection efficiency was about 24% for the SOX11-GFP-expressing plasmid. [file bcr3403-S21.JPEG]
