# Supplementary material for: Embryonic mammary signature subsets are activated in Brca1-/- and basal-like breast cancers
Source: Breast Cancer Res. 2013 Mar 18;15(2):R25. doi: 10.1186/bcr3403 (PMC3672751; doi:10.1186/bcr3403)
Supplement: Additional file 12 — Cluster-stability analysis of the hierarchic clustering of the embryonic mammary signature in breast cancer datasets by using the R-package pvclust. (A) Cluster-stability analysis; 55 of the 57 basal-like genes are in the left cluster, and the two major clusters are significantly different. (B) Cluster-stability analysis of the hierarchic clustering of the nonproliferative embryonic mammary signature in the UNC337 breast cancer samples. (C) Cluster-stability analysis of the hierarchic clustering of the nonproliferative embryonic mammary signature in the NKI295 breast cancer samples. [file bcr3403-S12.PDF]

A

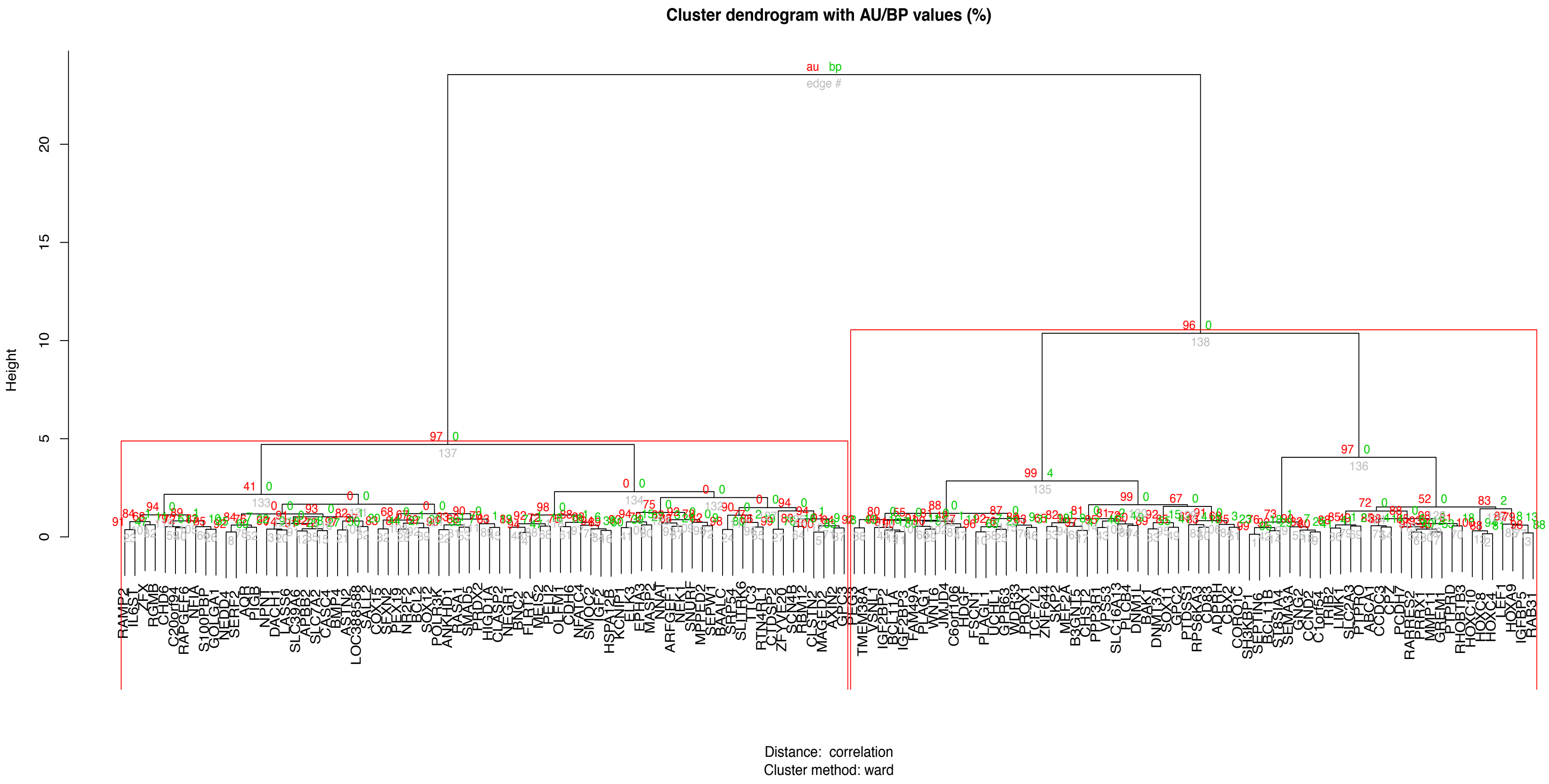
Cluster dendrogram with AU/BP values (%)
au bp
edge #
Height
0
5
10
15
20
Distance: correlation
Cluster method: ward

B

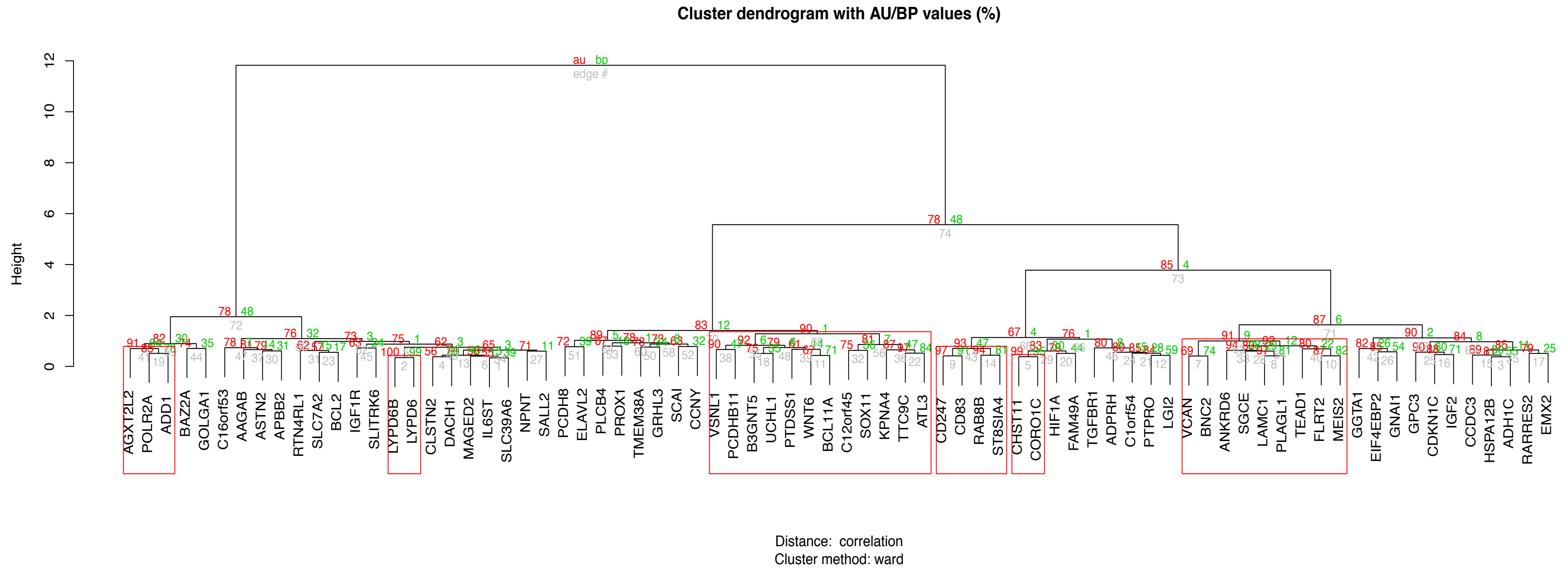

Non-basal clusters were less stable when the non-proliferative embryonic mammary gene signature was used to cluster the UNC337 dataset.

C

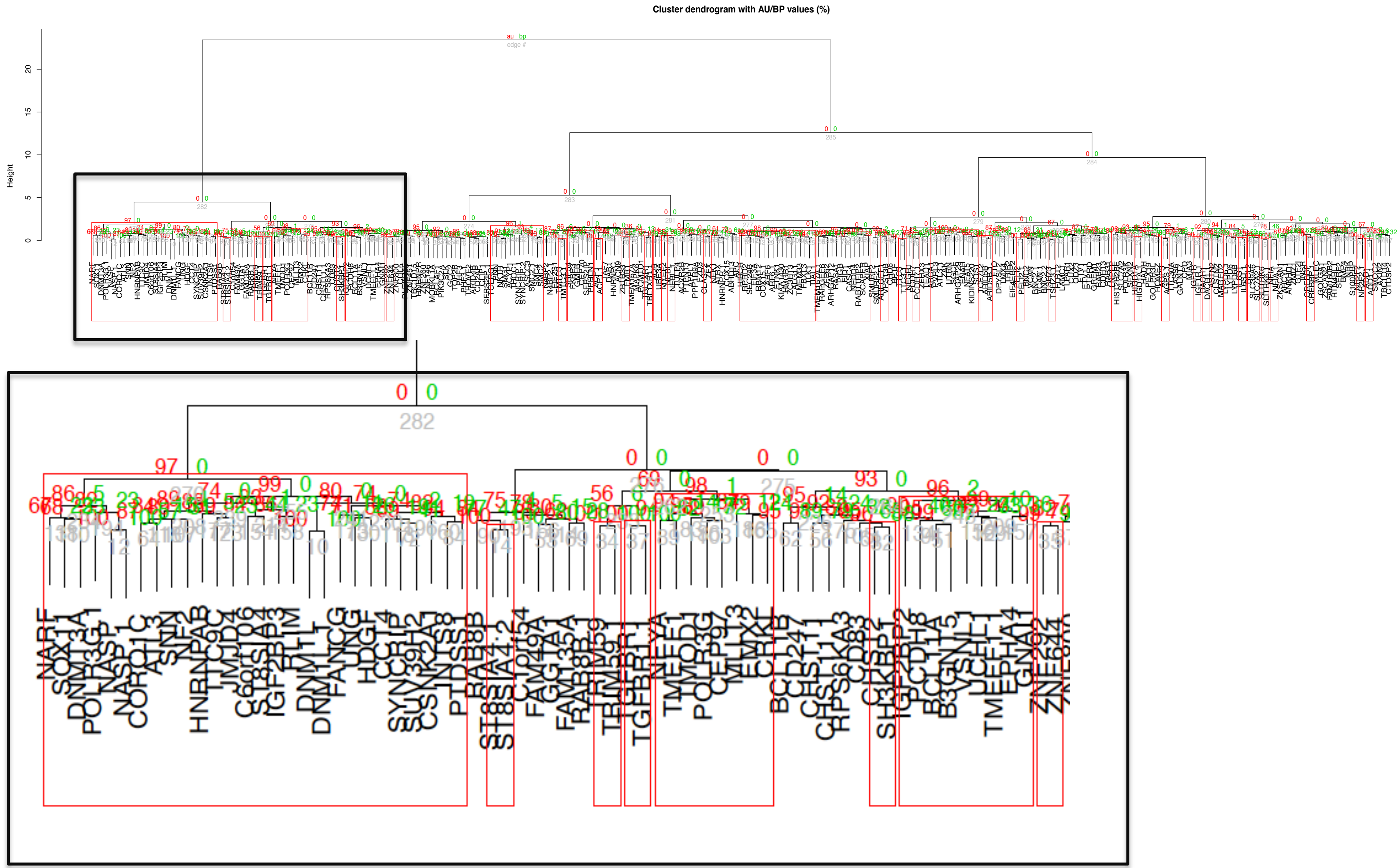

No significant difference between the two major clusters is observed in the NKI295 dataset, when the non-proliferative embryonic mammary gene signature was used to cluster them.
