# Supplementary material for: Embryonic mammary signature subsets are activated in Brca1-/- and basal-like breast cancers
Source: Breast Cancer Res. 2013 Mar 18;15(2):R25. doi: 10.1186/bcr3403 (PMC3672751; doi:10.1186/bcr3403)

ASPM

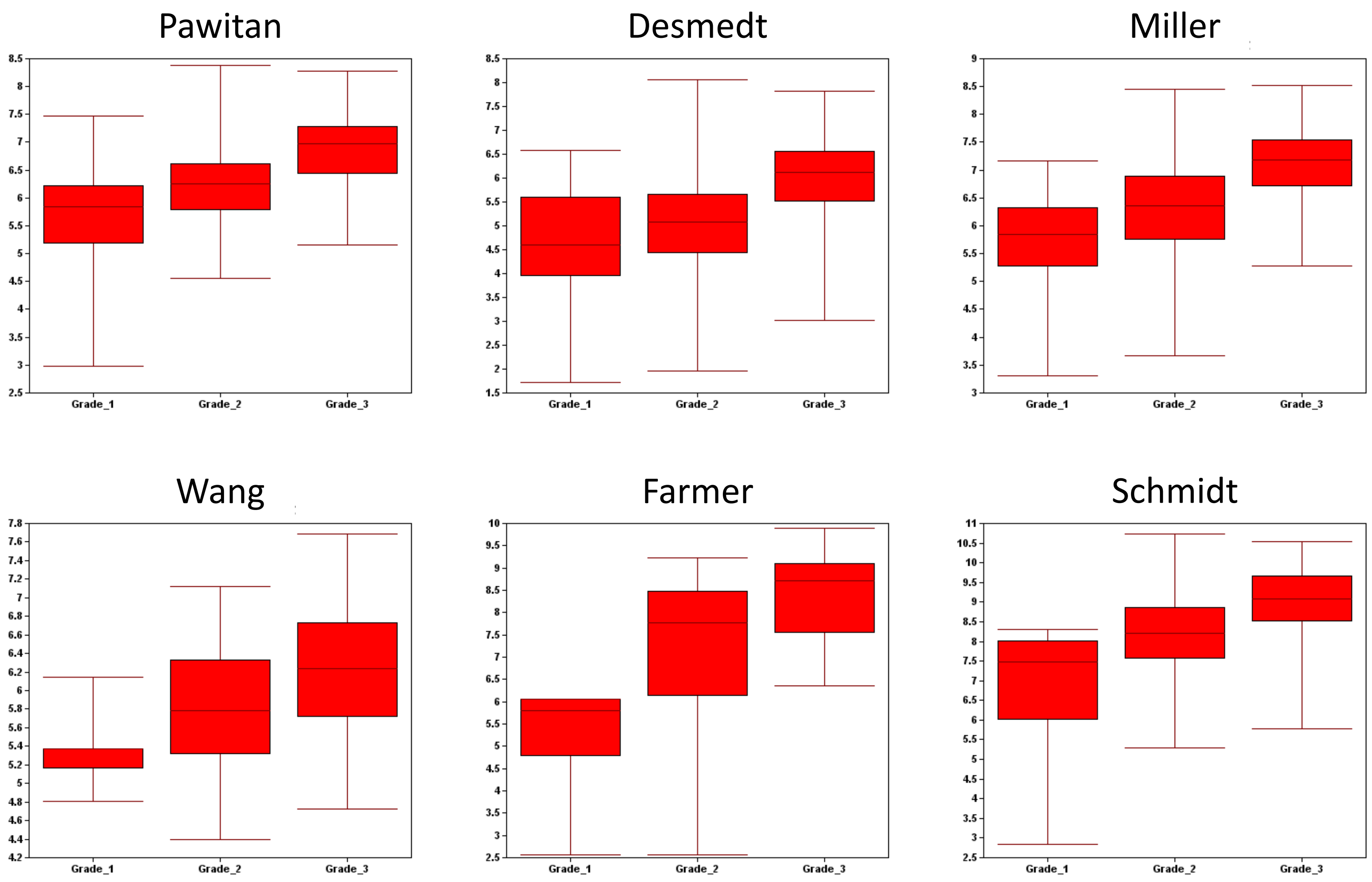
Pawitan
Desmedt
Miller
Wang
Farmer
Schmidt
Grade_1
Grade_2
Grade_3

CENPE

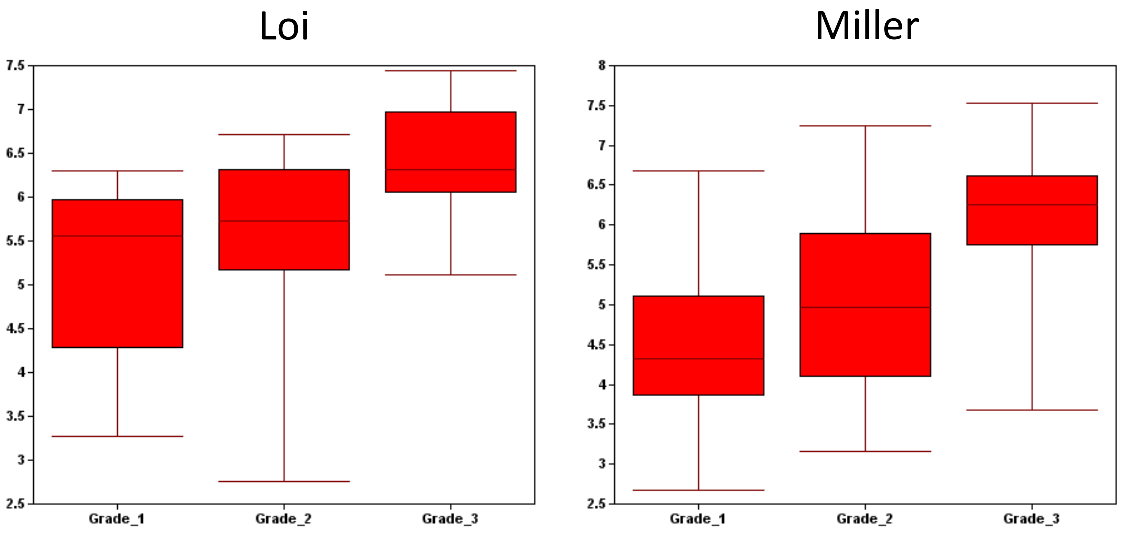
Loi
7.5
7
6.5
6
5.5
5
4.5
4
3.5
3
2.5
Grade_1
Grade_2
Grade_3
Miller
8
7.5
7
6.5
6
5.5
5
4.5
4
3.5
3
2.5
Grade_1
Grade_2
Grade_3

KIF11

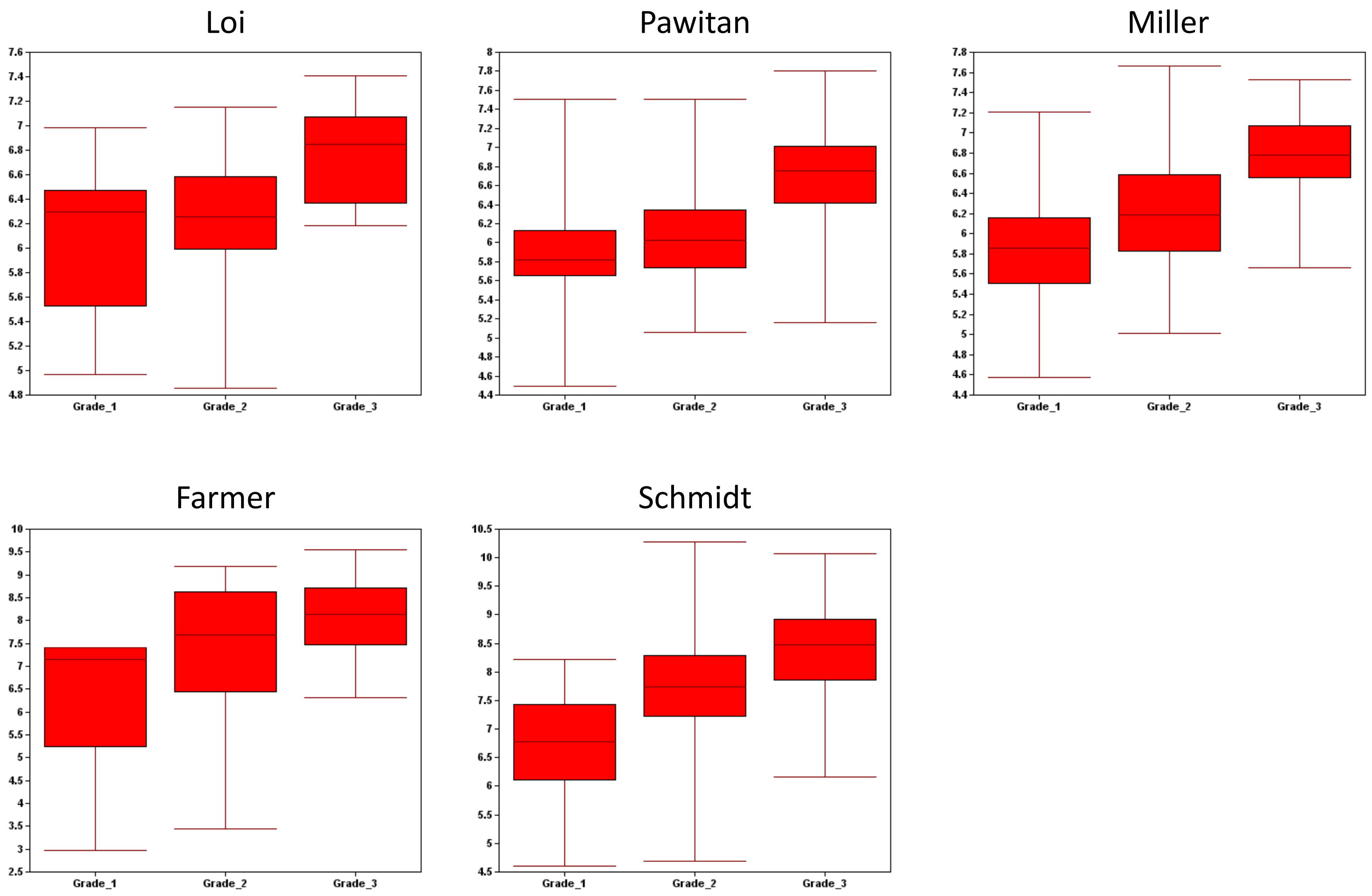

KIF20

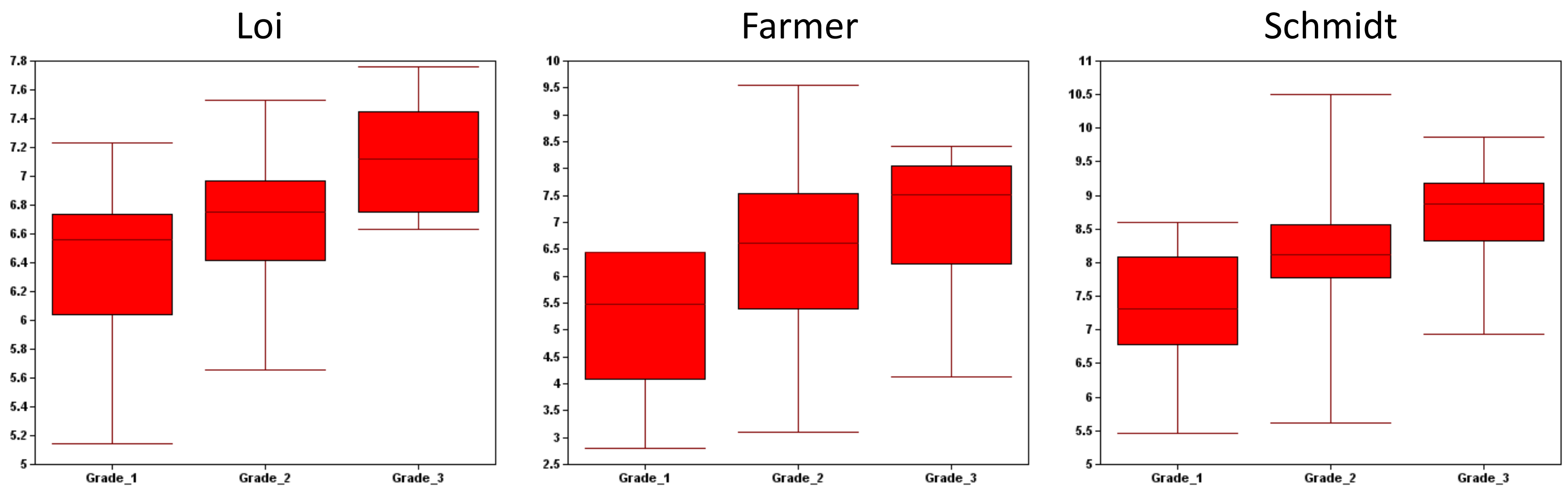
Loi
7.8
7.6
7.4
7.2
7
6.8
6.6
6.4
6.2
6
5.8
5.6
5.4
5.2
5
Grade_1
Grade_2
Grade_3
Farmer
10
9.5
9
8.5
8
7.5
7
6.5
6
5.5
5
4.5
4
3.5
3
2.5
Grade_1
Grade_2
Grade_3
Schmidt
11
10.5
10
9.5
9
8.5
8
7.5
7
6.5
6
5.5
5
Grade_1
Grade_2
Grade_3

SOX11

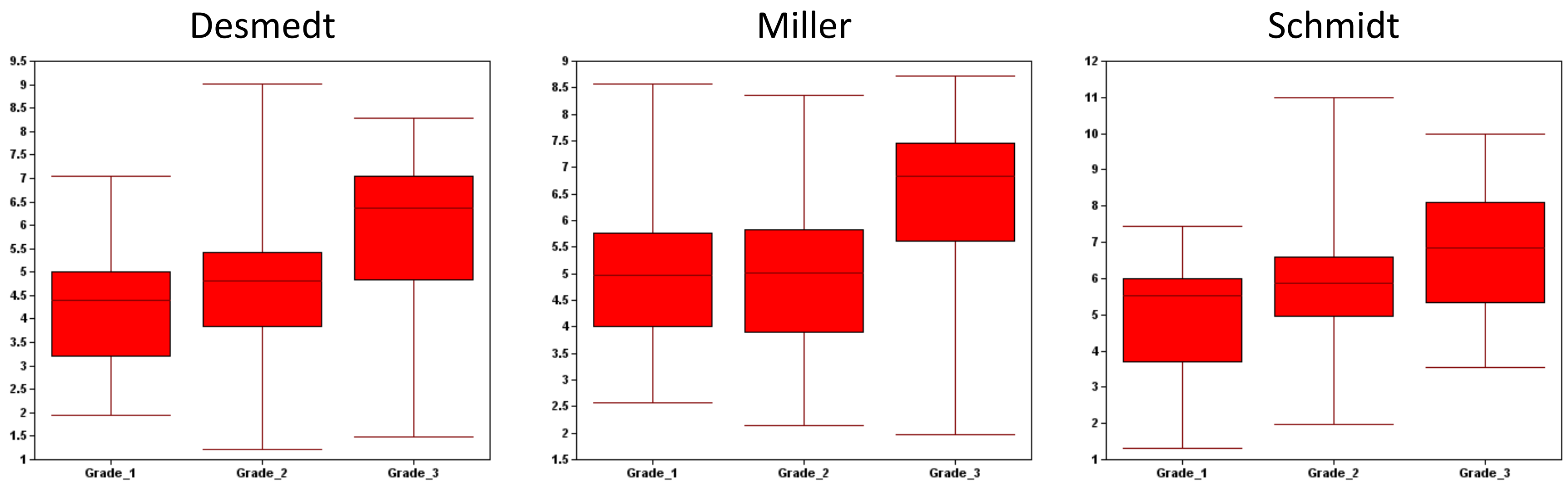

TPX2

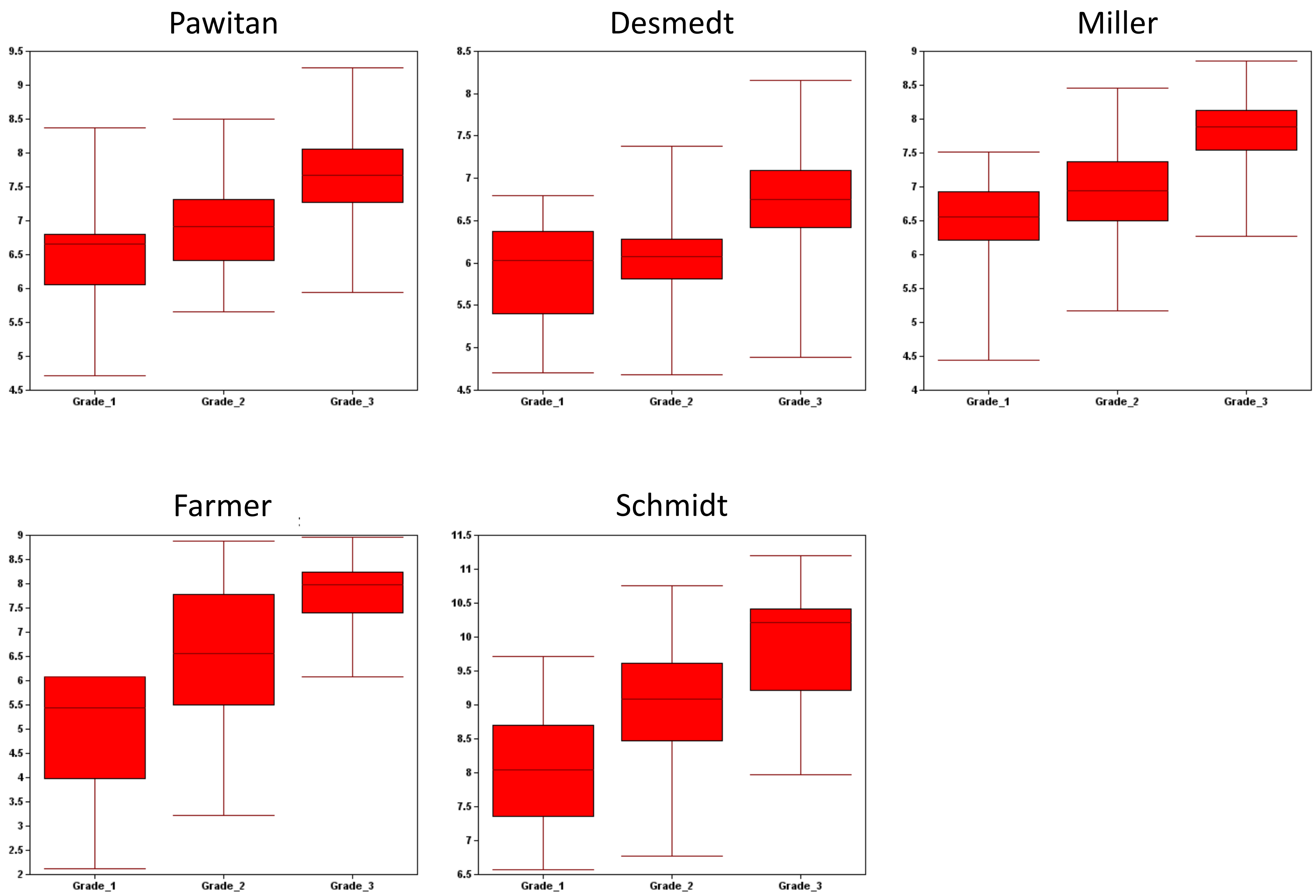
Pawitan
Grade_1
Grade_2
Grade_3
Desmedt
Grade_1
Grade_2
Grade_3
Miller
Grade_1
Grade_2
Grade_3
Farmer
Grade_1
Grade_2
Grade_3
Schmidt
Grade_1
Grade_2
Grade_3

TRIP13

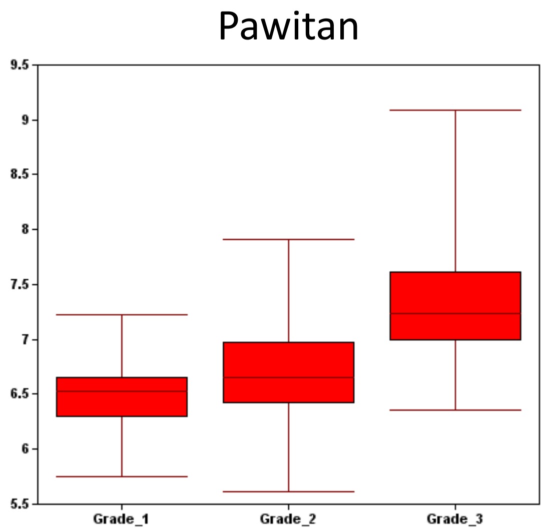

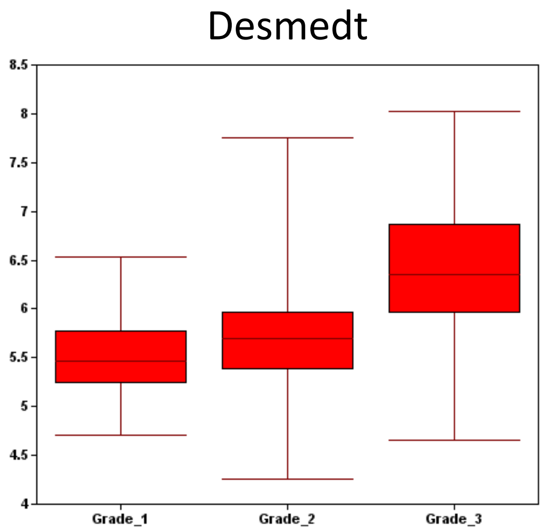

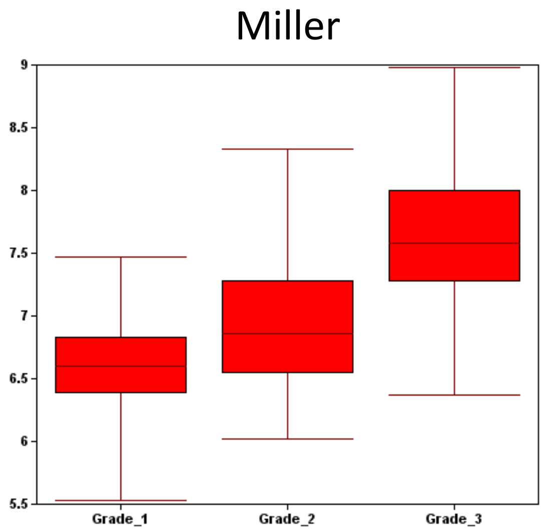

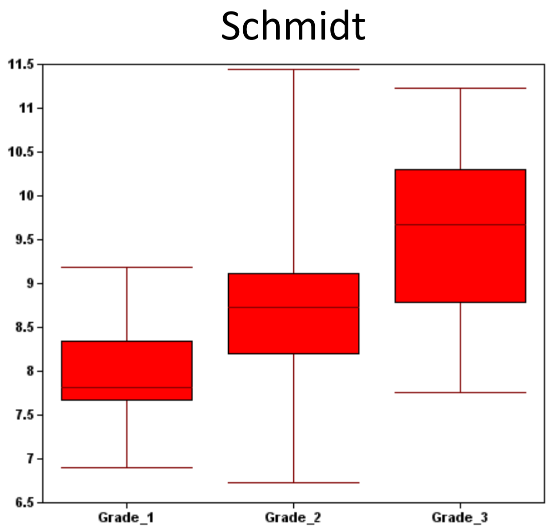

SAM results For Grade

	Loi	Pawitan	Desmedt	Miller	Wang	Farmer	Schmidt
SOX11	no Data	No Change	Change	Change	no Data	no Data	Change
ACN9	Not matched	Not matched	Not matched	Not matched	Not matched	Not matched	Not matched
UCHL1	no Data	no Data	No Change	No Change	no Data	no Data	No Change
BCL11A	no Data	No Change	no Data	No Change	No Change	no Data	Change
ASPM	no Data	Change	Change	Change	Change	Change	Change
TPX2	no Data	Change	Change	Change	No Change	Change	Change
KIF20A	change	No Change	No Change	No Change	No Change	Change	Change
TRIP13	No Change	Change	Change	Change	No Change	No Change	Change
KIF11	Change	Change	no Data	Change	no Data	Change	Change
VSNL1	no Data	no Data	no Data	no Data	no Data	no Data	No Change
FAM60A	no Data	no Data	No Change	No Change	no Data	no Data	No Change
CENPE	Change	no Data	no Data	Change	no Data	no Data	No Change

Breast Cancer Subtypes:
ASPM

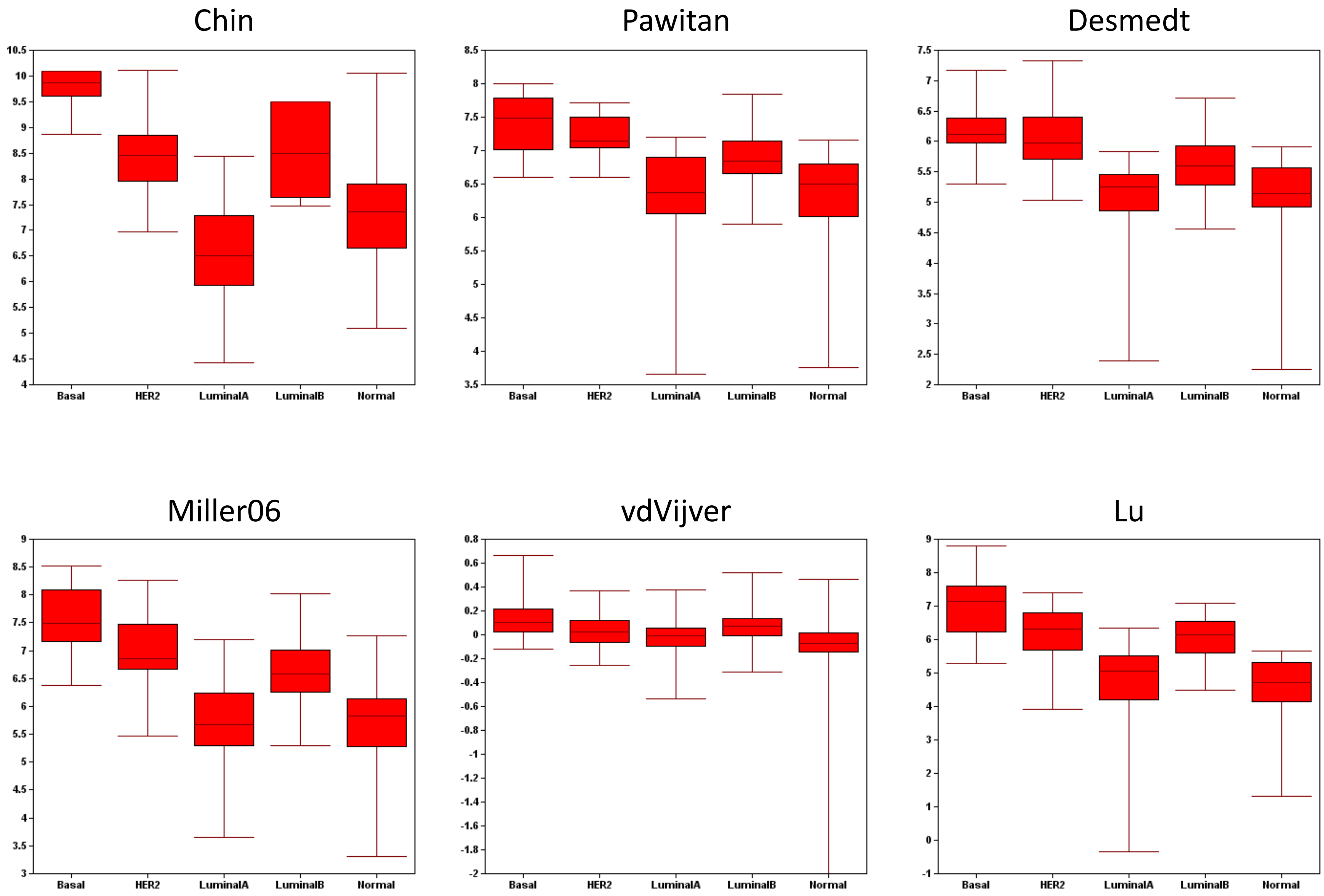

Breast Cancer Subtypes:
BCL11A

Chin

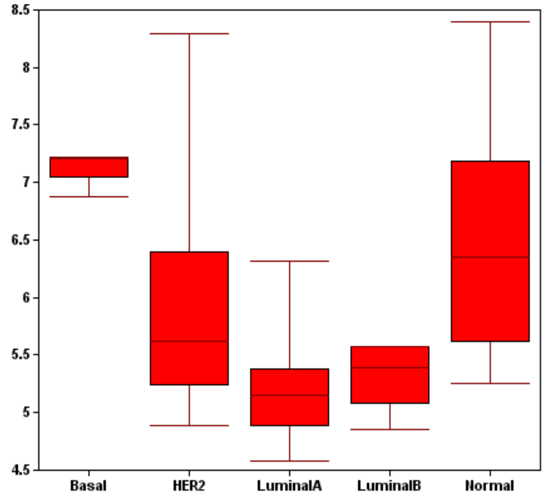

Pawitan

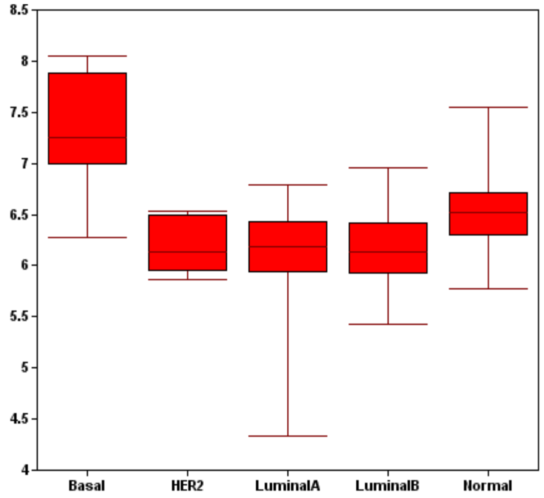

Miller 05

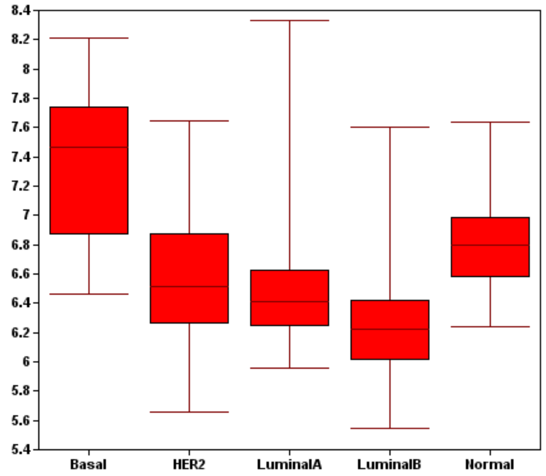

vdVijver

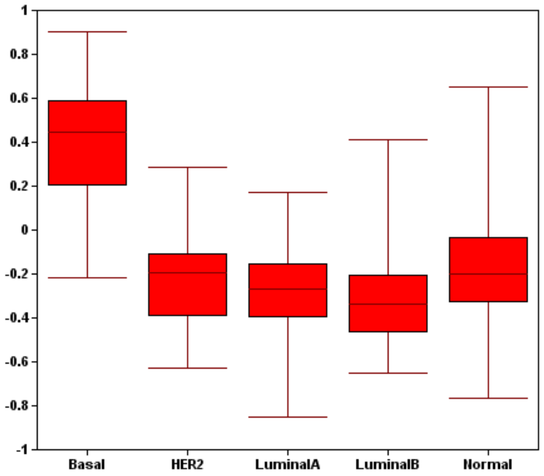

Lu

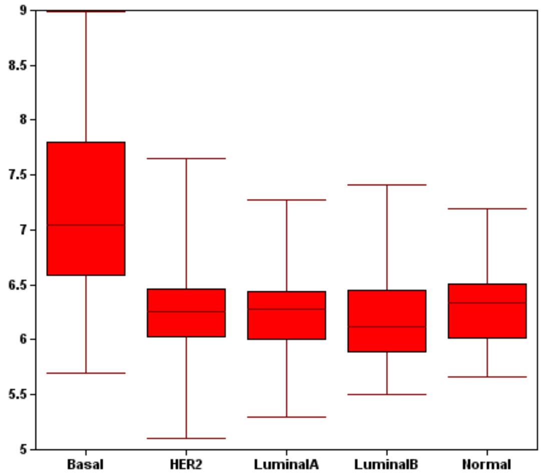

Breast Cancer Subtypes:
CENPE

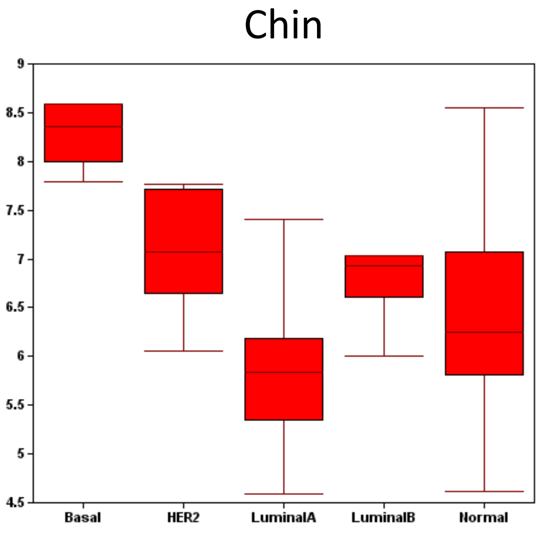

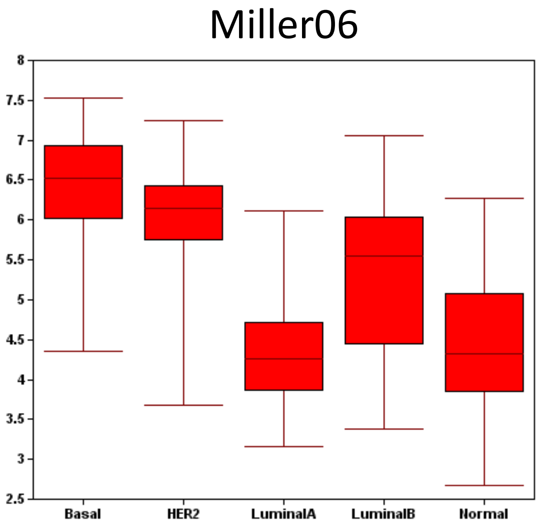

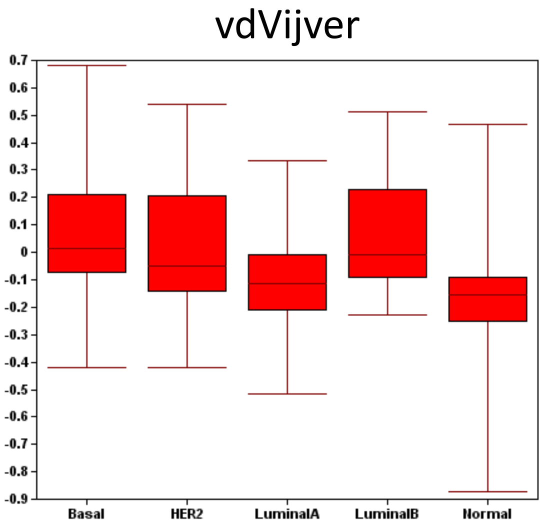

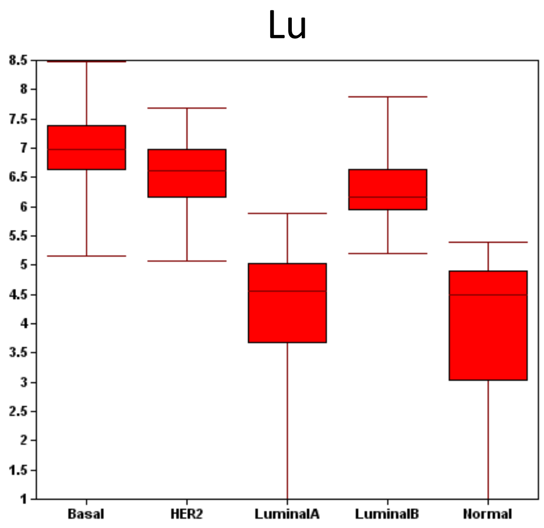

Breast Cancer Subtypes:
FAM60A

Chin

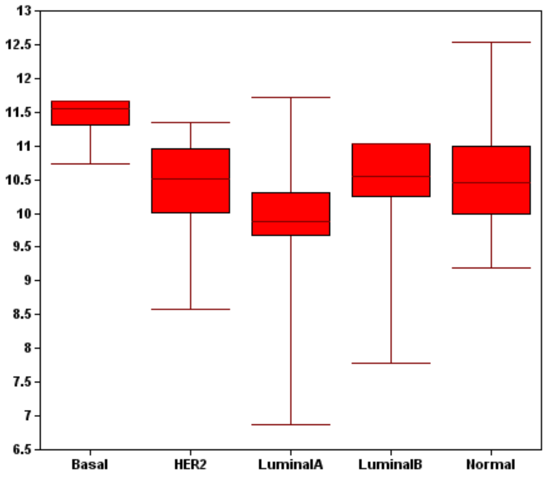

Desmedt

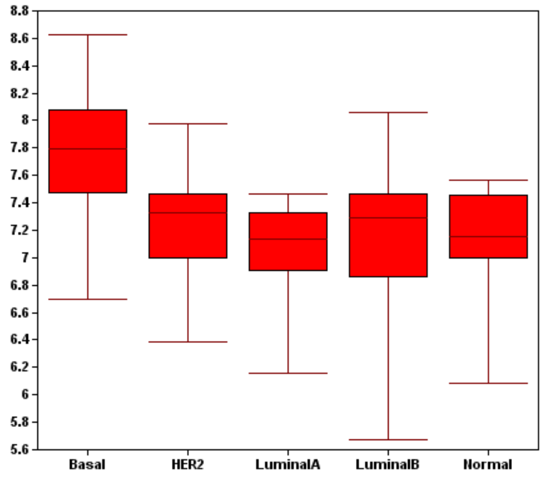

Miller06

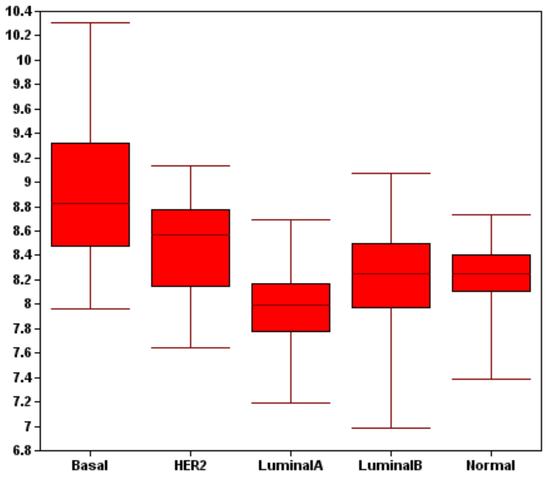

Lu

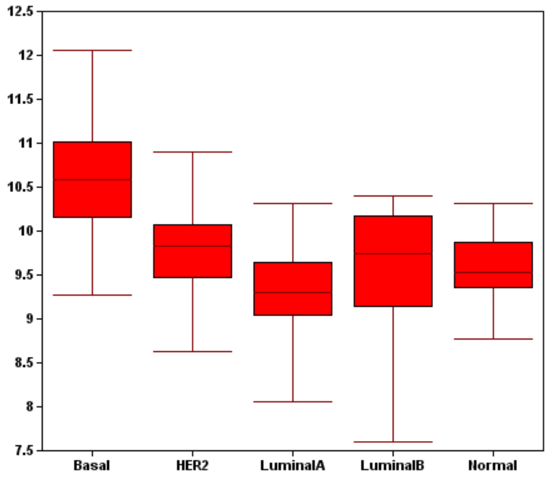

Breast Cancer Subtypes:
KIF11

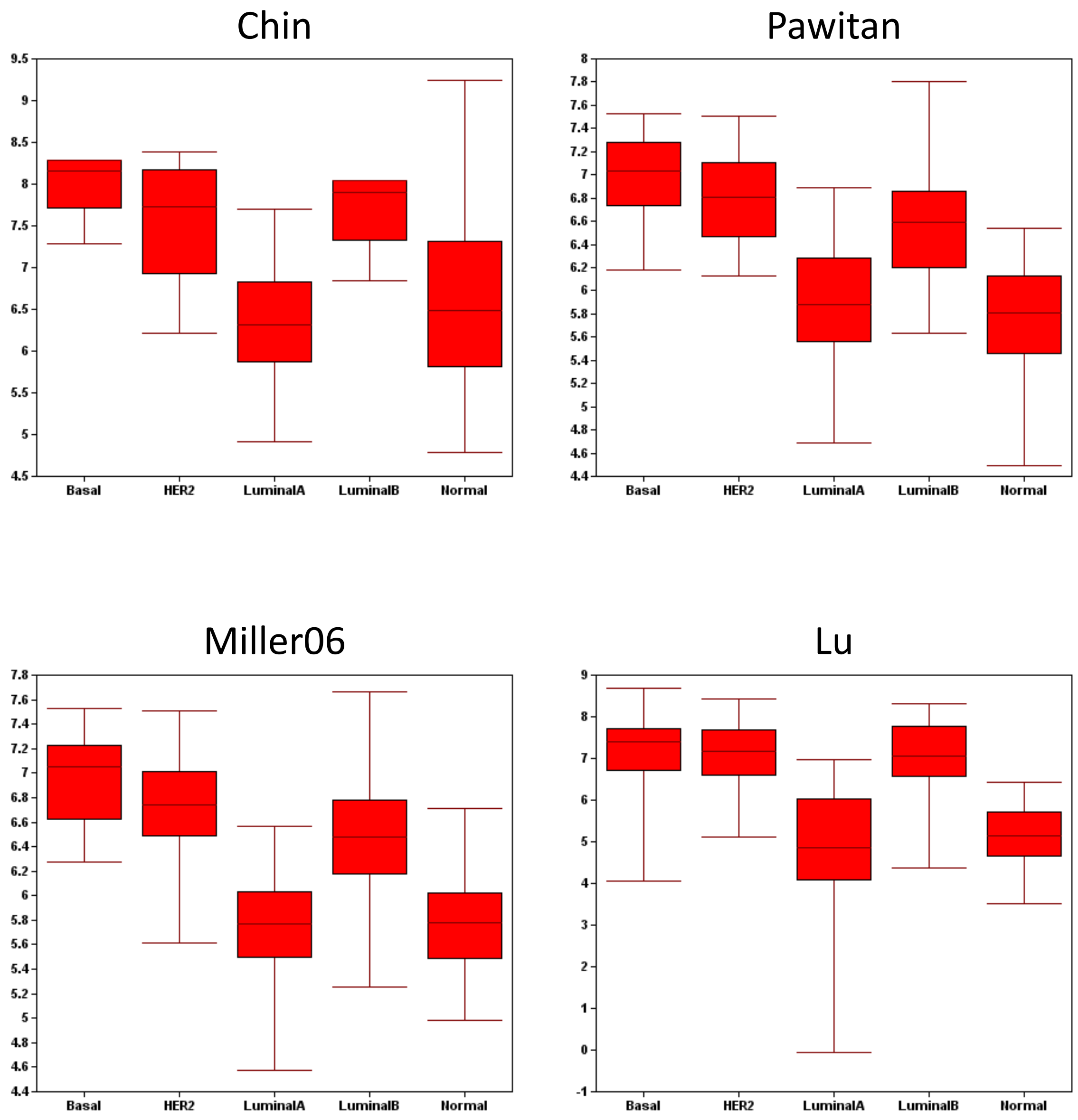

Breast Cancer Subtypes:
KIF20A

Chin

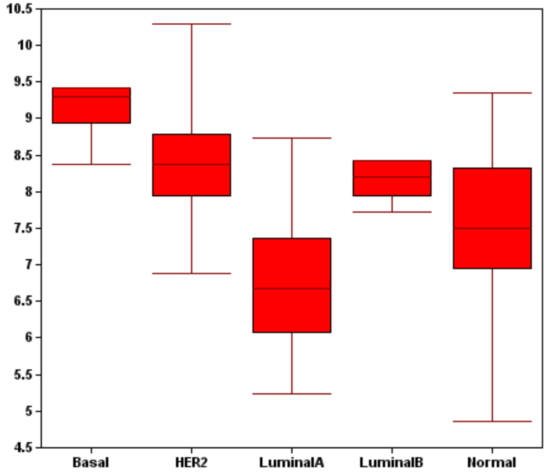

Pawitan

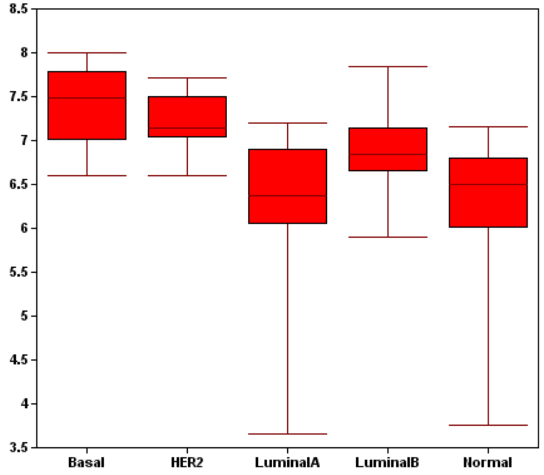

Desmedt

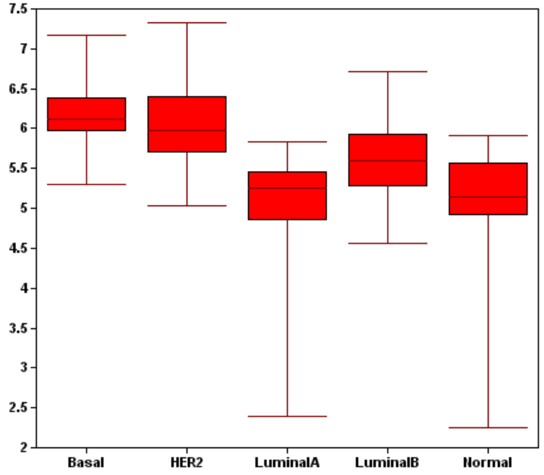

Miller06

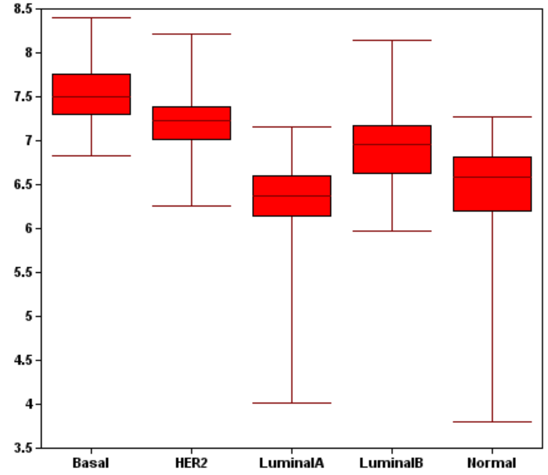

Lu

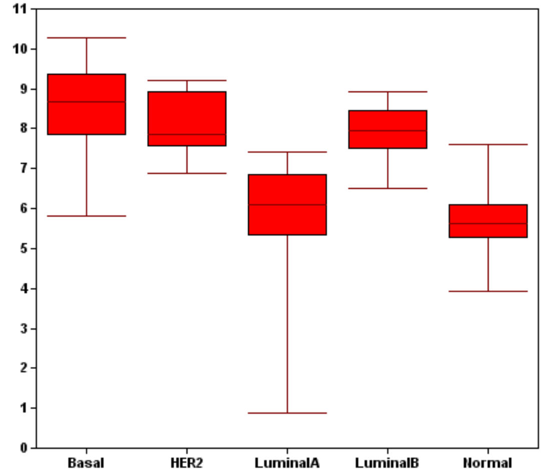

Breast Cancer Subtypes:
SOX11

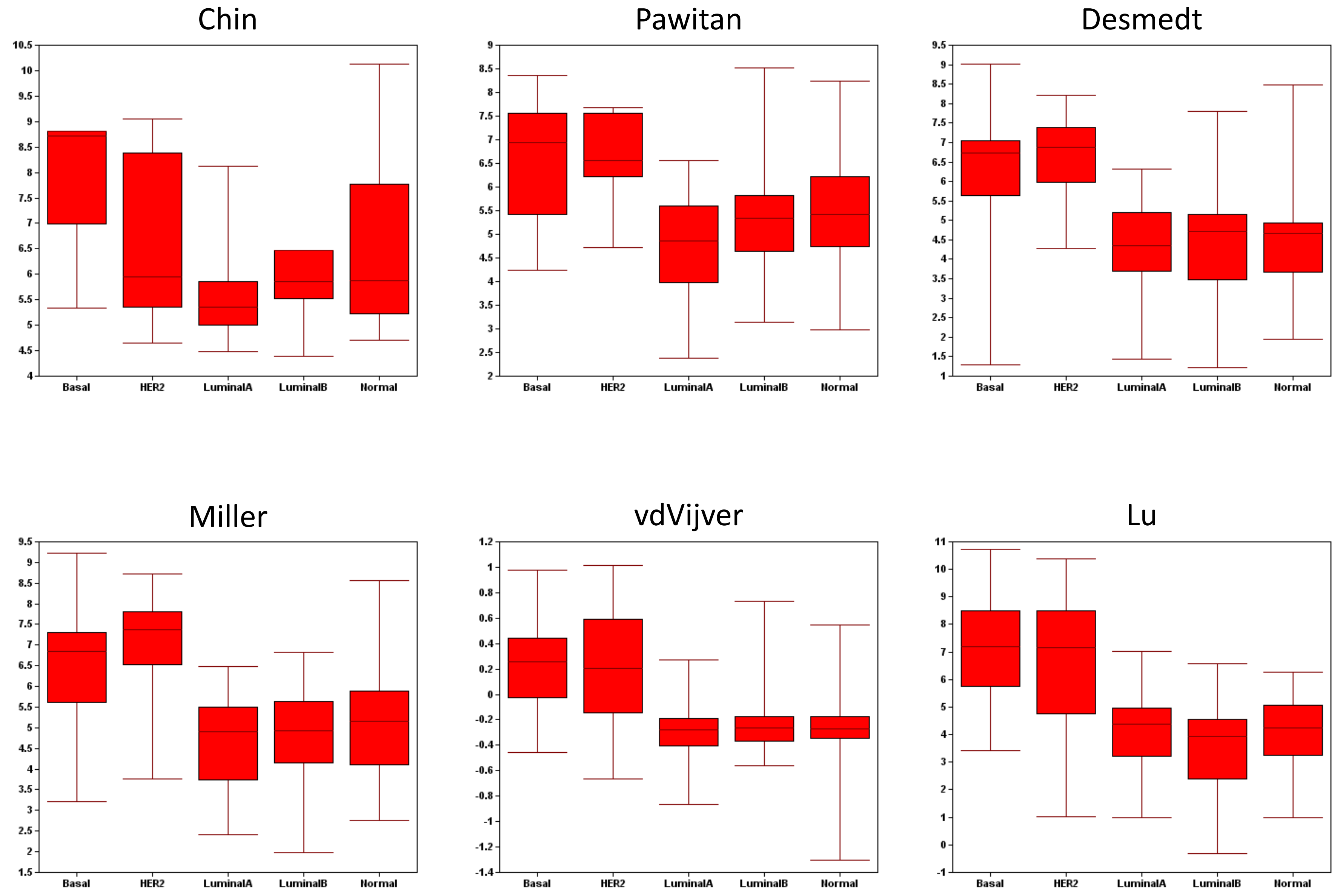

Breast Cancer Subtypes:
TPX2

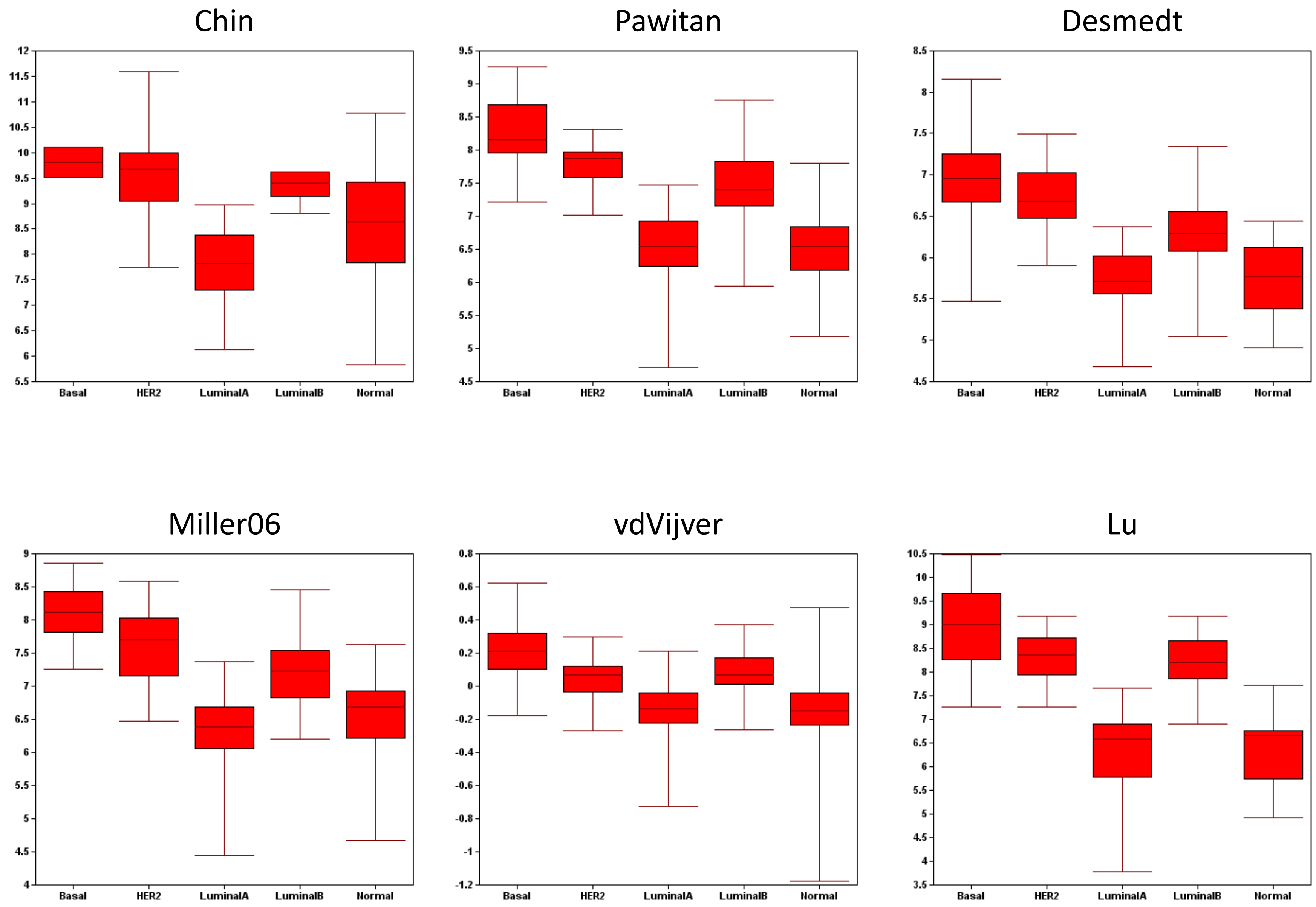

Breast Cancer Subtypes:
TRIP13

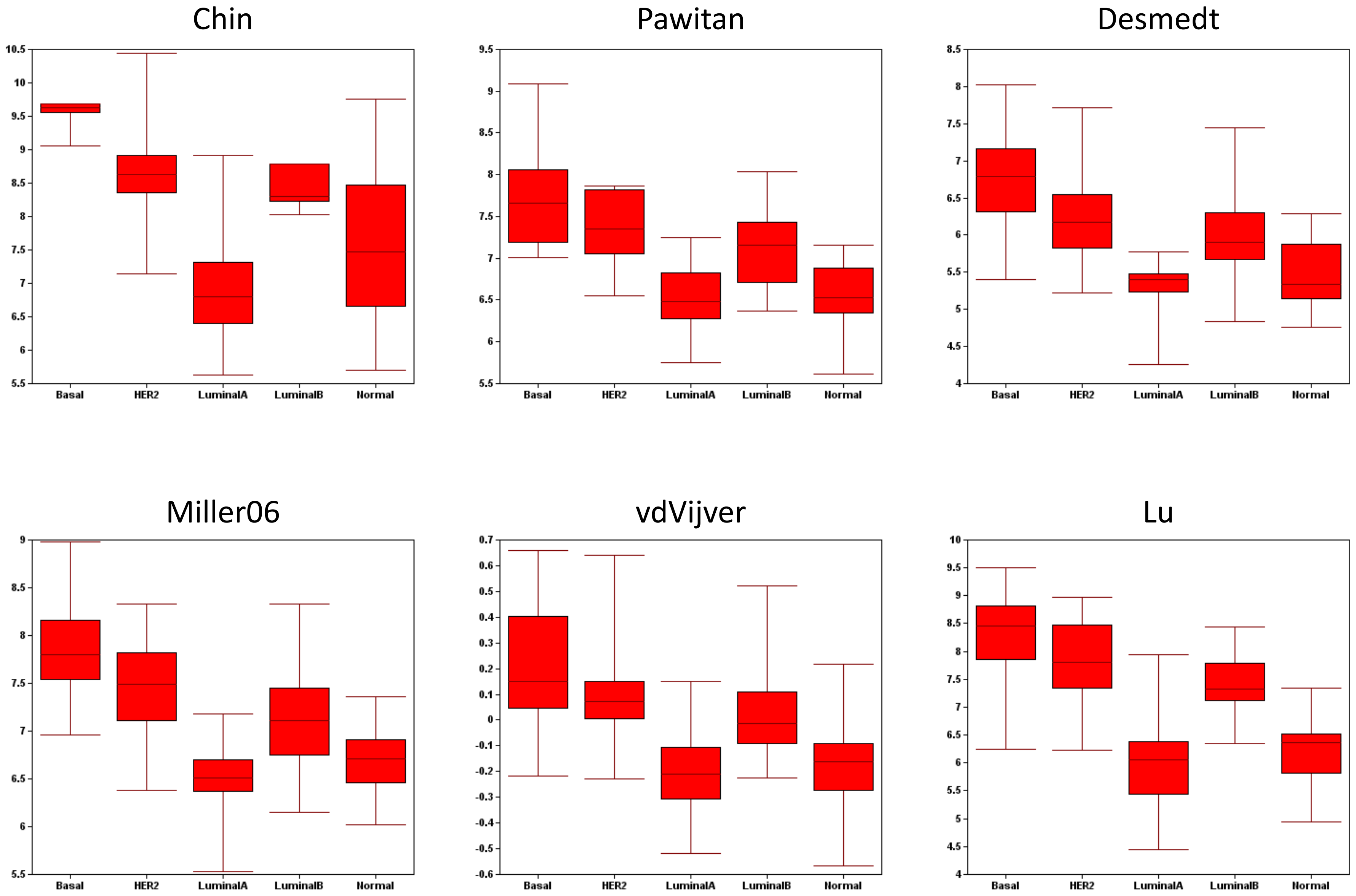

Breast Cancer Subtypes:
UCHL1

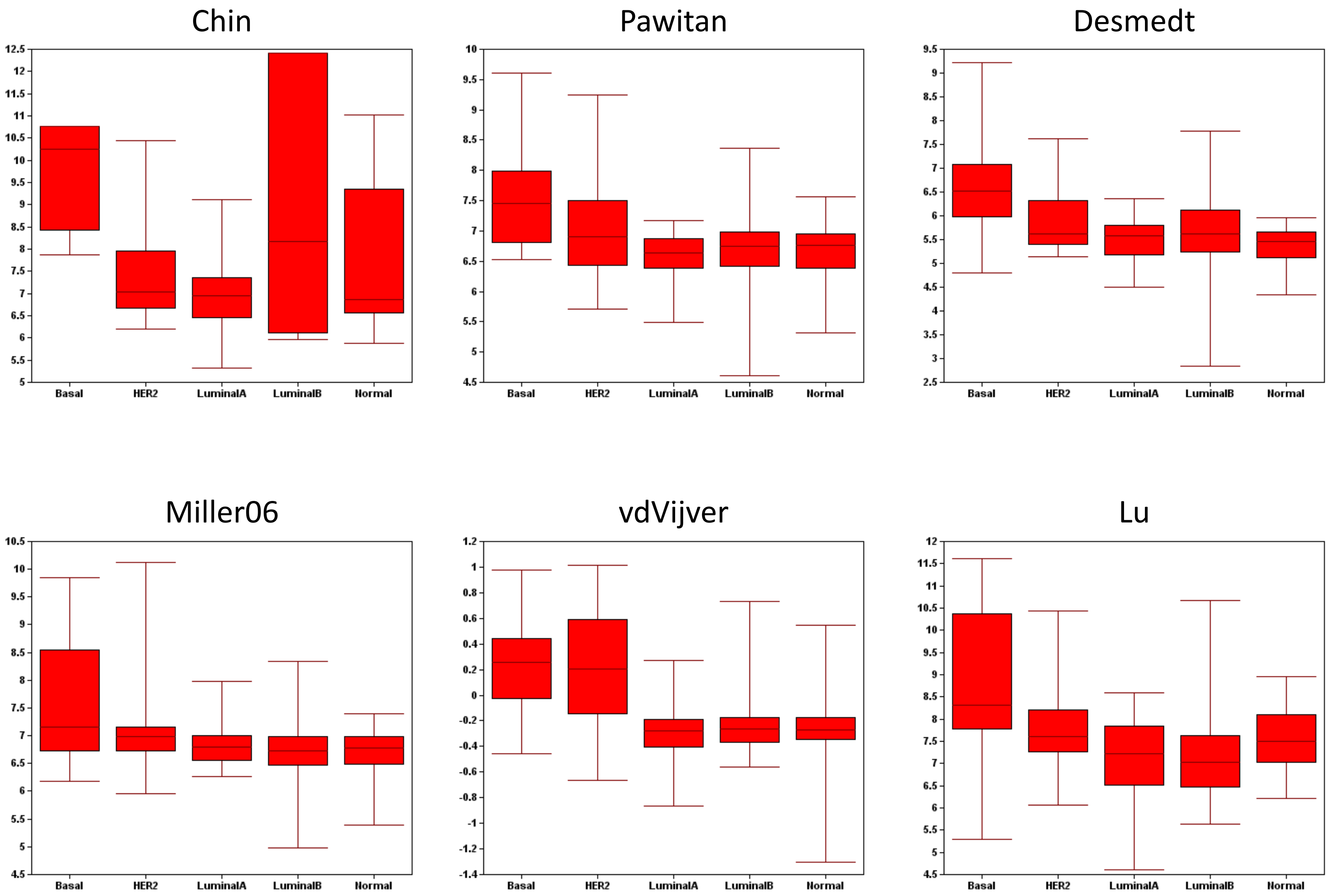

Supplement: Additional file 19 — Significance analysis of microarray analysis of the expression of core network of tumor-associated embryonic genes according to tumor grade and tumor subtype. [file bcr3403-S19.PDF]
